# Maternal diabetes alters transcriptional programs in the developing embryo

**DOI:** 10.1186/1471-2164-10-274

**Published:** 2009-06-18

**Authors:** Gabriela Pavlinkova, J Michael Salbaum, Claudia Kappen

**Affiliations:** 1 Department of Genetics, Cell Biology and Anatomy, University of Nebraska Medical Center, Omaha, NE 68198-5455, USA; 2 Department of Pediatrics, University of Nebraska Medical Center, Omaha, NE 68198-5455, USA; 3 Munroe-Meyer Institute for Genetics and Rehabilitation, University of Nebraska Medical Center, Omaha, NE 68198-5455, USA; 4 Department of Maternal Biology, Pennington Biomedical Research Center, 6400 Perkins Road, Baton Rouge, LA 70808, USA; 5 Department of Regulation of Gene Expression, Pennington Biomedical Research Center, 6400 Perkins Road, Baton Rouge, LA 70808, USA; 6 Laboratory of Molecular Pathogenetics, Institute of Biotechnology of the Academy of Sciences of the Czech Republic, Videnska 1083, Prague 4, CZ-14220, Czech Republic

## Abstract

**Background:**

Maternal diabetes is a well-known risk factor for birth defects, such as heart defects and neural tube defects. The causative molecular mechanisms in the developing embryo are currently unknown, and the pathogenesis of developmental abnormalities during diabetic pregnancy is not well understood. We hypothesized that the developmental defects are due to alterations in critical developmental pathways, possibly as a result of altered gene expression. We here report results from gene expression profiling of exposed embryos from a mouse diabetes model.

**Results:**

In comparison to normal embryos at mid-gestation, we find significantly altered gene expression levels in diabetes-exposed embryos. Independent validation of altered expression was obtained by quantitative Real Time Polymerase Chain Reaction. Sequence motifs in the promoters of diabetes-affected genes suggest potential binding of transcription factors that are involved in responses to oxidative stress and/or to hypoxia, two conditions known to be associated with diabetic pregnancies. Functional annotation shows that a sixth of the de-regulated genes have known developmental phenotypes in mouse mutants. Over 30% of the genes we have identified encode transcription factors and chromatin modifying proteins or components of signaling pathways that impinge on transcription.

**Conclusion:**

Exposure to maternal diabetes during pregnancy alters transcriptional profiles in the developing embryo. The enrichment, within the set of de-regulated genes, of those encoding transcriptional regulatory molecules provides support for the hypothesis that maternal diabetes affects specific developmental programs.

## Background

Maternal diabetes disturbs embryonic development and can cause diabetic embryopathy, with cardiovascular malformations, neural tube defects and caudal dysgenesis as the most characteristic congenital malformations [1,2]. Diabetes-induced dysmorphologies have been ascribed to increased apoptosis [3,4], perturbation of prostaglandin synthesis and metabolism [5-7], deficiency in membrane lipids [8-11], and generally altered metabolism in the embryo [12]. Several studies have associated oxidative stress with the maternal diabetic condition, and the administration of anti-oxidants reduced the incidence of developmental defects in experimental models of intrauterine exposure to diabetes [5,13-19]. However, it is unclear how systemic metabolic disease results in particular developmental defects that are restricted to specific tissues in diabetic embryopathy [20].

Growing evidence suggests that maternal diabetes alters expression of developmental genes in the embryo, resulting in abnormal morphogenesis. Decreased expression of Pax3, a gene involved in neural tube defects [21,22], was found in embryos from diabetic mouse dams at gestation day 8.5, with neural tube defects evident by day 10.5 [4]. Pax3 deregulation, presumably through oxidative stress [23], is also associated with heart defects that involve neural crest cell derivatives [24]. We recently showed that Wnt signaling is affected in diabetes-exposed mouse embryos [25]. These findings support the notion that diabetic pregnancy leads to altered expression of molecules that play key roles in patterning and development of embryonic tissues, implicating altered transcriptional regulation as a potential pathogenic mechanism in diabetic embryopathy. In order to identify genes and pathways affected by maternal diabetes, we performed gene expression profiling of diabetes-exposed mouse embryos using oligonucleotide microarrays.

## Results and discussion

### Animal model of diabetic embryopathy

**Figure 1:**
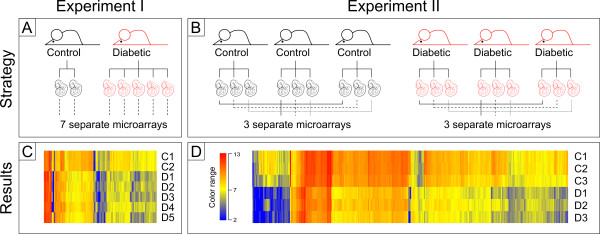
**Experimental approaches to determine gene expression profiles of normal and diabetes-exposed embryos**. Panels A and B depict the two independent microarray experiments. Panels C and D depict expression profiles where each colored vertical line represents the expression signal for one gene and row represent individual embryos (Exp. I) or samples (Exp. II). Red represents increased expression, blue reflects decreased expression, and intermediate colors represent minor changes (the color range was chosen along an arbitrary scale). Using a hierarchical clustering algorithm (with euclidean distance metric and centroid linkage rule, as implemented in GeneSpring), these graphic representations shows that expression profiles for embryos exposed to maternal diabetes differ considerably from control unexposed embryo profiles.

Mouse embryos were isolated from diabetic or control dams at embryonic day 10.5 (E10.5) because at this stage neural tube defects are easily detectable. The frequency of NTDs in diabetes-exposed embryos was approximately 17% (16/96 diabetes-exposed embryos) compared to 0% in normal pregnancies (0/220). Except where noted, no malformed embryos were used for gene expression studies. We found no significant differences in litter size or number of resorbed embryos between diabetic (n = 11) and non-diabetic pregnancies (n = 10; P = 0.07). We also did not detect any developmental delay in apparently unaffected diabetes-exposed embryos; their morphological appearance, i.e. features of brain development, limb development, and somite numbers were commensurate with developmental age (Kruger et al., manuscript in preparation). All experiments used the FVB inbred strain. We here report the results from two independent expression profiling experiments (Figure 1), using individual embryo samples in Experiment I and and a pooling strategy in Experiment II.

### Gene expression profiling in embryos exposed to diabetes

In Experiment I, we surveyed the expression profiles of 2 control and 5 diabetes-exposed embryos. In an initial comparison of expression levels between control and diabetes-exposed embryos, 302 probe sets passed the "fold-change>2" criterion, and their expression profiles were visualized using hierarchical clustering (Figure 1, Panel C). Control embryos exhibited profiles similar to each other, and differences to the expression profiles of individual diabetes-exposed embryos are visually obvious. These results support the hypothesis that maternal diabetes affects gene expression in the exposed embryos.

This survey covered about 14000 genes, and after application of the analysis criteria (see Methods for details), we identified 126 genes (~1% of the total) with expression levels that were changed in diabetes-exposed embryos by more than 2-fold relative to controls (Table 1; for full gene names and accession numbers, see Additional File 1). An additional 378 genes displayed expression differences between 1.5- and 2-fold (data not shown). The majority (83%) of the 126 genes we identified were expressed at lower levels in diabetes-exposed compared to control embryos, and this was reflected in the larger dataset as well (72.8% of the additional 378 genes were decreased in expression). This decreased gene expression was not the result of developmental delay, since the morphology of diabetes-exposed embryos was stage-appropriate.

**Table 1:** Genes affected by maternal diabetes classified by cellular function. see Additional file 1

Functional category	# of genes	% of total	Gene Symbol
Transcription factors	15	12	Bcl11a, Cited4, Creb1, Crsp2, Hif1a, Klf9, Lin28, Nsd1, Rb1cc1, Rnf14, Zfa, Zfp60, Zfp294, Zfp385, 2610020O08Rik
DNA-binding/chromatin	8	6	Atrx, Baz1b, Exod1, Hist1h2bc, Hmga1, Msl31, Setdb1, Top2b
Signal transduction	15	12	Ap1g1, Arid4b, Gad1, Grb10, Mapk10, Phip, Pik3c2a, Pkia, Ptp4a3, Ptprs, Rabgap1, Rp2h, Stam2, Ywhag, Zcsl3
Cell surface, incl. receptors	13	10	Agtr2, Aplp2, Cxadr, Efnb2, Epha3, Ghr, Gpr65, Il6st, Itgav, Pdgfra, Ptprk, Sema3a, Tgfbr1
Extracellular matrix/adhesion	9	7	Adam10, Ctse, Hs6st2, Ndst1, Ogt, Pcdh18, Pxn, Sel1h, Twsg1
Cytoskeleton/microtubules	10	8	Dcx, Dnm1l, Epb4.1l2, Gmfb, Kif11, Mtap2, Sncg, Tubb2b, Tubb2c, Vcl
RNA-binding	7	6	Arl5a, Dcp1a, Pabpc1, Rnpc2, Rod1, Sfrs2, Syncrip
Transporter/channels	9	7	Abcb7, Aqr, Cacna2d1, Mbtps1, Slc2a1, Slc16a3, Slc25a22, Stx17, Tm9sf3
Metabolism/enzymes	6	5	Aldh18a1, Blvrb, Gmpr, Pfkl, Tnks2, Upp1
Lipid metabolism	6	5	Etnk1, Hdlbp, Scd2, Sgpl1, Sptlc1, Stom
Metal-ion homeostasis	3	2	LOC669660, Mt2, Tfrc
Protein catabolism	9	7	Arih2, Nedd4, Supt16h, Usp7, Usp12, Eif3s10, Gopc, Lin7c, Vps35
Cell cycle/apoptosis	3	2	Api5, Birc4, Kras
Other	6	5	Dysf, Ivns1abp, Pelp1, Plekha5, Trim2, Trim44
Unknown	7	6	Heatr1, BC067396, 1300007C21Rik, 6330503C03Rik, 6330527O06Rik, 6330578E17Rik, LOC640370

total	126	100	

### Validation of microarray by quantitative real-time PCR

Changes in gene expression detected by microarray were validated by quantitative real time PCR (Q-RT-PCR) for selected genes with potential relevance to diabetes or embryonic development (Table 2). Embryo samples were from different pregnancies than those employed for the microarray studies, and only embryos were used that appeared morphologically normal. Of all genes assayed, 16 exhibited differential expression in the Q-RT-PCR assay, confirming the microarray results in independent embryo samples. Three genes exhibited no differences, and seven genes were differentially expressed in both assays (P < 0.05); however, the change occurred in opposite directions. The discrepancies were traced back to (i) annotation problems: Hmga1, Lin28, Phip, (ii) different length of 3'UTR sequences where location of the microarray probe would not query all transcripts arising from the respective genes: Sema3a, Rod1, Slc2a1, or (iii) alternative splicing: Ogt, Tfrc [26], Creb1 [27]. The Q-RT-PCR assays were designed to specifically amplify a region of the transcript different from that covered by the microarray probe in order to obtain an independent measurement. For the majority of genes we tested, the independent assay confirms the initial finding that expression levels are altered in embryos exposed to maternal diabetes.

**Table 2:** Validation of microarray results by quantitative RT-PCR.

	**Microarray**		**qRT-PCR**	
Gene Symbol	Fold change	t-test (p-value)	Fold change	t-test (p-value)
Adam10*	-2.37	0.0024	-1.56	0.035
Api5*	-2.30	0.0004	-1.33	0.007
Atrx	-2.15	0.0016	-1.33	0.046
Baz1b*	-2.38	0.0012	-1.58	0.017
Cxadr*	-2.00	0.0034	-1.38	0.0002
Dcx*	-3.05	0.0001	-2.20	<0.0001
Efnb2*	-2.04	0.0437	-1.72	0.010
Hif1a	-2.52	0.0041	-1.49	0.011
Il6st	-2.28	0.0198	-1.77	0.039
Mt2	3.45	0.0053	2.16	0.027
Mtap2	-3.02	0.0000	-1.98	0.0002
Pcdh18*	-5.77	0.0017	-1.35	<0.0001
Pdgfra1	-2.01	0.0100	-1.42	0.018
Tgfβ r1	-3.26	0.0002	-1.89	0.010
Twsg1	-4.91	0.0002	-1.89	0.005
Vcl	-2.37	0.0020	-2.03	0.002
				
Hmga1	2.13	0.0060	-2.14	<0.0001
Lin28*	2.00	0.0182	-1.30	0.032
Ogt	2.52	0.0310	-1.51	0.019
Slc2a1	2.15	0.0252	-1.68	0.012
Phip	-2.76	0.0006	1.49	0.011
Rod1*	-2.32	0.0004	1.20	0.041
Sema3a	-2.05	0.0003	1.74	0.024
				
Creb1	-3.33	0.0007	1.05	ns
Cited4*	2.11	0.0026	-1.10	ns
Tfrc*	-2.20	0.0023	1.11	ns

For each gene, the amplification rate was calculated from the linear range of the reaction, and diabetes-exposed embryos (n = 9 from 4 litters) were compared to 4 pools (4 litters) of mRNA from control embryos. In reactions for genes marked with *, the control group consisted of mRNA from individual embryos (n = 6 from 4 litters). All embryos were isolated at E10.5 and were morphologically normal. Negative values for "fold-change" indicate reduction of expression.

We cannot formally exclude altered mRNA stability as a factor causing the observed changes in mRNA levels, but it would be difficult to explain how the stability of relatively few transcripts could be altered in a selective fashion. Rather, we find that many transcription factor genes are down-regulated in their expression in diabetes-exposed embryos, and this trend is also reflected in the group of genes with 1.5- to 2-fold differences in expression; most likely therefore, the observed lower levels of gene expression are due to diminished or deregulated transcription.

### Molecular classification of genes altered in response to maternal diabetes

**Figure 2:**
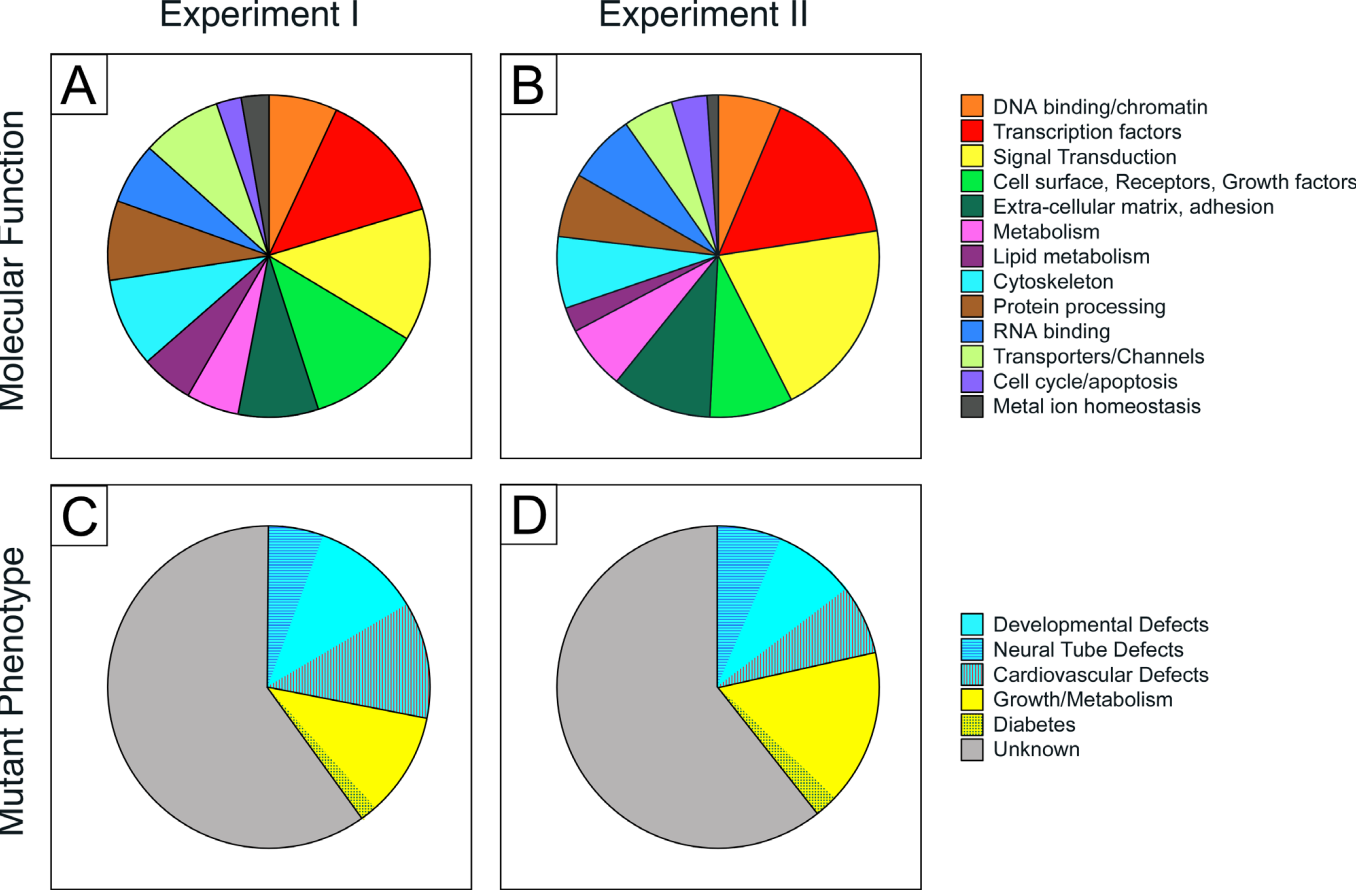
**Expression of Hif1α during mouse embryogenesis**. Quantitative RT-PCR for Hif1α at various stages of development normalized to expression levels of Pole4. n = number of individual embryos tested.

The biological roles of many products encoded by the 126 diabetes-affected genes are known (Table 1 and Figure 2, Panel A). Most intriguingly, the largest functional category is comprised by transcription factors (15/126) and DNA-binding molecules known to affect transcriptional regulation (8/126), with both categories together comprising 18% of the identified genes. Thus, relative to the 7% of genes in the mouse genome that encode transcription factors [28], we found transcription regulatory genes highly overrepresented among our diabetes-affected genes. This is also reflected in enrichment of this category in DAVID annotation . A combined 22% of the genes encode cell surface receptors (10%) and signal transduction molecules (12%) that ultimately converge on transcription. The transcription factor Pax3, which was previously identified as affected by maternal diabetes [4] was not represented on the microarray, but was changed as expected when assayed by Q-RT-PCR [25]. These results indicate that transcription factors and signaling molecules are prominent targets for perturbation by maternal diabetes, and that altered transcriptional regulation plays a major role in the response of embryos to intrauterine exposure to diabetic conditions.

### Transcription regulation

If maternal diabetes deregulates cohorts of genes in the developing embryo through shared pathways of transcriptional regulation, one would expect (i) occurrence of common transcription factor binding sites (TFBS) in regulatory regions associated with multiple genes, and (ii) overrepresentation of such TFBS relative to other genes in the genome. We therefore analyzed the promoter sequences (5kb upstream of the transcription start) of our diabetes-affected genes for the presence of TFBS. As expected for genes with mostly broad expression patterns [29], we found a diverse set of conserved motifs in the upstream regions of these genes (see Additional file 2). Intriguingly, there was prominent over-representation of binding sites for the transcription factors FOXO1 and FOXO4 (-Log(p) = 12.416 and -Log(p) = 10.544, respectively), which are known to be involved in the response to oxidative stress, and for HIF1 (-Log(p) = 10.219), which is involved in the response to hypoxia. Out of 109 genes for which results were returned, FOXO1 and FOXO4 sites were enriched in 68% of the genes. NRF2 motifs were found in 76 promoters, further supporting the notion that oxidative stress may be involved in the response of diabetes-exposed embryos. HIF1 motifs were enriched in promoter regions of 22 genes (20.2%). These TFBS occur in combinations with sites for other transcription factors with a known role in responses to hypoxia, such as ATF4, E2F1 and E2F4, EGR1, ETS1, IRF1, NfkappaB, SOX9, SP3, and XBP1 (see Additional file 2 for references). Given the proposed role of oxidative stress and hypoxia in the pathogenesis of diabetic embryopathy [5,6,14,17,18,30], it is striking that 97% of the genes affected by maternal diabetes carry in their upstream regions potential binding sites for transcription factors that are involved in responses to oxidative stress and hypoxia.

**Figure 3:**
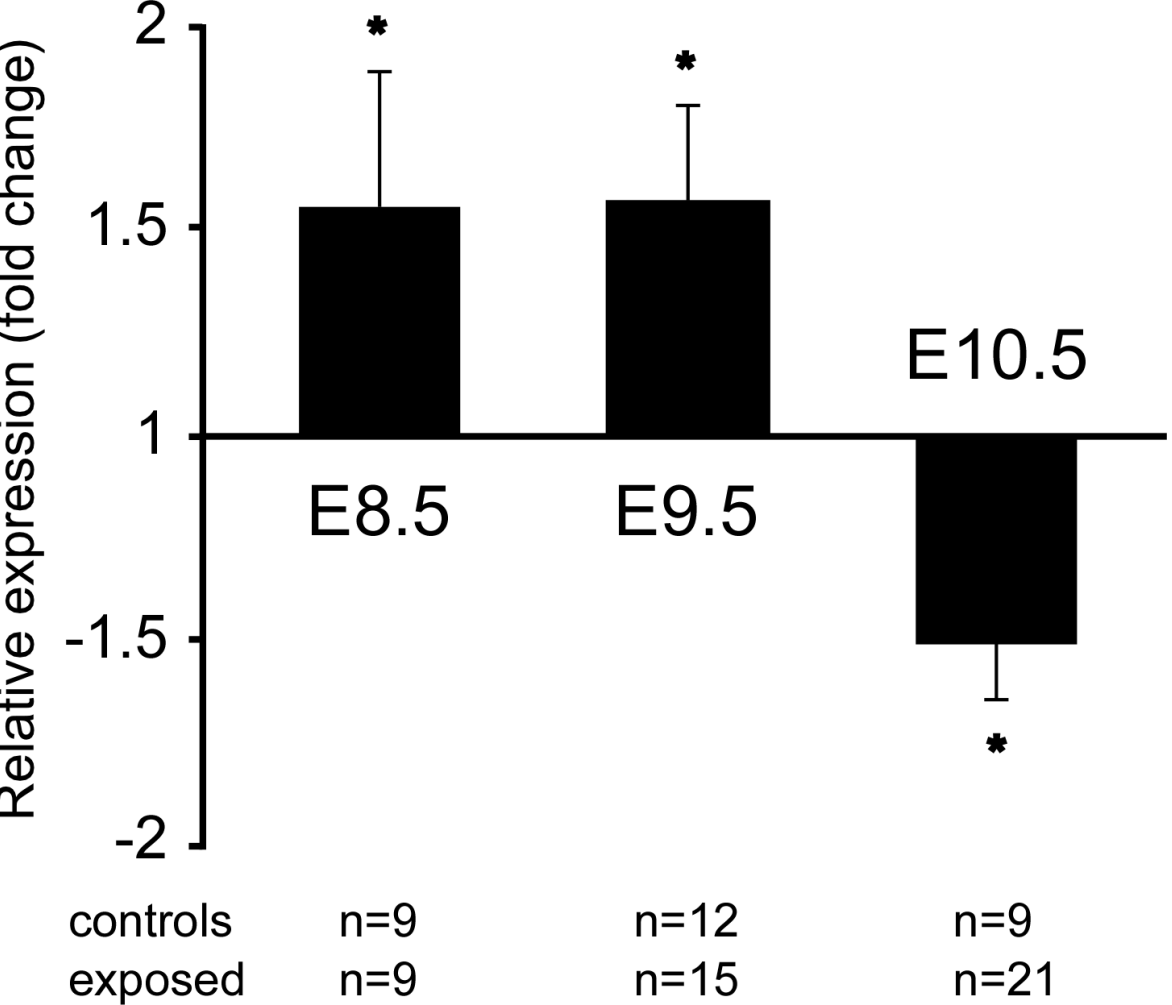
**Classification of diabetes-affected genes by molecular function and function in vivo**. Panel A depicts the representation of molecular classes of encoded products for the diabetes-affected genes identified in Experiment I. Panel B depicts the representation of molecular classes for the diabetes-affected genes identified in Experiment II. Genes encoding products with unknown function were omitted. Panel C depicts in vivo phenotypes based upon MGI annotation for genes identified in Experiment I; Panel D depicts in vivo phenotypes for genes in Experiment II. Only knockout phenotypes (null and conditional) were included.

Both conditions have been reported to be associated with diabetic pregnancy [14,31], and would predict activation of hypoxia-regulated pathways in the embryonic response to diabetic conditions. Paradoxically, the expression of Hif1α, a key regulator of embryonic responses to hypoxia [32], was reduced in our diabetes-exposed embryos at E10.5. In a post-hoc analysis of our microarray data with specific focus on HIF1 target genes [33], 22 HIF1 targets showed altered expression (fold-change >1.5) and passed one of the t-tests; 9 genes passed both statistical filters (details, see Additional file 3). Twenty HIF1-regulated genes exhibited increased expression in diabetes-exposed embryos, possibly reflecting an embryonic response to increased hypoxia. Further support for this idea comes from Hif1α message levels that are increased in diabetes-exposed embryos at E8.5 and E9.5 (Figure 3), and this increased Hif1α expression could be responsible for the increased expression of HIF target genes at E10.5. Together, these results implicate oxidative stress and hypoxia pathways in deregulated gene expression in diabetes-exposed embryos and identify the molecular targets of these pathways.

### Functional roles of genes deregulated by maternal diabetes

Our identification of genes whose expression is affected by exposure to maternal diabetes suggests that those genes could be involved in the developmental defects in diabetic pregnancies. This notion is supported by qualitative expression information (MGI) for 69 of these genes, with 62 detected in the embryonic CNS, and 35 in the embryonic cardiovascular system. With both CNS and heart frequently being affected in diabetic embryopathy, genes with abnormal expression in these tissues might contribute to pathogenesis of birth defects.

Functional data also support this hypothesis: most remarkably, about one fourth of the diabetes-affected genes are known genes for which a functional role in embryonic development was established experimentally: in knockout mutant mice, 35 of the genes we identified have been shown, by genetics, to be required for mouse embryonic development (Table 3 and Figure 3, Panel C; references are given in Supplementary Table 4). This implies that these genes, under conditions of maternal diabetes but in the absence of genetic alterations, are subject to gene-environment interactions and respond to the intra-uterine environment of a diabetic pregnancy. Deficiencies in 15 of our genes (Agtr2, Cxadr, Dysf, Hif1a, Il6st, Itgav, Pdgfra, Pxn, Sema3a, Tgfbr1, Vcl, Adam10, Epha3, Efnb2, and Sfrs2) have been shown to cause cardiovascular defects or disease in mouse models. In humans, the risk for congenital heart disease is 2.8 times higher in infants born to diabetic mothers compared to the offspring of non-diabetic mothers [2] with higher odds ratios for specific malformations [34,35]. In light of this, it is possible that down-regulation by maternal diabetes of one or more of the genes we discovered could contribute to abnormalities of the heart [36].

**Table 3:** In vivo function of genes affected in diabetes exposed embryos.

Function	#	GeneSymbol
metabolic/growth defect	15	Ap1g1, Aplp2, Ghr, Grb10, Hmga1, Mapk10, Mbtps1, Mtap2, Nedd4, Ptprs, Scd2, Tnks2, Top2b, Upp1, Zfp385

diabetes	2	Ghr, Hmga1

embryonic	35	Abcb7, Adam10, Agtr2, Ap1g1, Aplp2, Bcl11a, Creb1, Cxadr, Dcx, Efnb2, Epha3, Gad1, Grb10, Hif1a, Il6st, Itgav, Kras, Mbtps1, Ndst1, Nedd4, Nsd1, Ogt, Pdgfra, Ptprs, Pxn, Scd2, Sema3a, Setdb1, Sfrs2, Slc2a1/Glut1AS^a^,, Tfrc, Tgfbr1, Top2b, Twsg1, Vcl

cardiovascular	15	Adam10, Agtr2, Cxadr, Dysf, Efnb2, Epha3, Hif1a, Il6st, Itgav, Pdgfra, Pxn, Sema3a, Sfrs2, Tgfbr1, Vcl

neural tube defects	7	Adam 10, Hif1a, Pdgfra, Tfrc, Tgfbr, Twsg1, Vcl

Information was obtained from GO annotations and hand curated with input from MGI and PubMed sources. All results are from knockout models, except for: ^a^) Glut 1 antisense transgenic mice. References are provided in Additional file 4.

**Table 4:** Wnt-pathway genes affected by maternal diabetes.

	Control		Exp. II					
	Mean	(+/-SD)	Mean	(+/-SD)	p-value	fold-change	Gene Symbol	Gene Title
1450007_at	602	(± 141)	76	(± 23)	0.0031	-7.93	1500003O03Rik	similar to EF-hand Ca2+ binding protein p22
1450056_at	423	(± 43)	103	(± 42)	0.0008	-4.12	Apc	adenomatosis polyposis coli
1455231_s_at	326	(± 71)	97	(± 3)	0.0052	-3.36	Apc2	adenomatosis polyposis coli 2
1426966_at	1257	(± 52)	530	(± 52)	6.6^-5^	-2.37	Axin1	axin 1
1444031_at	17	(± 5)	47	(± 6)	0.0028	2.80	Camk2d	calcium/calmodulin-dependent protein kinase II, delta
1417176_at	3354	(± 833)	1102	(± 385)	0.0132	-3.04	Csnk1e	casein kinase 1, epsilon
1422887_a_at	3473	(± 351)	1625	(± 222)	0.0015	-2.14	Ctbp2	C-terminal binding protein 2
1430533_a_at	3643	(± 841)	333	(± 352)	0.0033	-10.95	Ctnnb1	beta-catenin
1458662_at	167	(± 35)	75	(± 18)	0.0152	-2.23	Daam1	dishevelled associated activator of morphogenesis 1
1450978_at	965	(± 42)	430	(± 40)	9.0^-5^	-2.24	Dvl1	dishevelled homolog 1
1417207_at	993	(± 91)	327	(± 46)	0.0004	-3.04	Dvl2	dishevelled homolog 2
1455220_at	181	(± 12)	76	(± 12)	0.0004	-2.39	Frat2	frequently rearranged in advanced T-cell lymphomas 2
1437284_at	2115	(± 66)	965	(± 32)	1.1^-5^	-2.19	Fzd1	frizzled homolog 1
1418532_at	1720	(± 389)	551	(± 193)	0.0096	-3.12	Fzd2	frizzled homolog 2
1450044_at	2329	(± 163)	884	(± 174)	0.0005	-2.63	Fzd7	frizzled homolog 7
1423348_at	419	(± 33)	188	(± 35)	0.0011	-2.23	Fzd8	frizzled homolog 8
1427529_at	136	(± 24)	57	(± 21)	0.0125	-2.40	Fzd9	frizzled homolog 9
1455689_at	485	(± 124)	204	(± 22)	0.0182	-2.37	Fzd10	frizzled homolog 10
1451020_at	434	(± 144)	156	(± 28)	0.0303	-2.79	Gsk3b	glycogen synthase kinase 3 beta
1417409_at	1585	(± 117)	664	(± 114)	0.0006	-2.39	Jun	Jun oncogene
1425795_a_at	1069	(± 122)	423	(± 92)	0.0018	-2.53	Map3k7	mitogen activated protein kinase kinase kinase 7
1452497_a_at	192	(± 85)	39	(± 4)	0.0353	-4.97	Nfatc3	nuclear factor of activated T-cells, calcineurin-dependent 3
1423379_at	497	(± 87)	81	(± 9)	0.0012	-6.17	Nfatc4	nuclear factor of activated T-cells, calcineurin-dependent 4
1419466_at	353	(± 31)	166	(± 5)	0.0005	-2.13	Nkd2	naked cuticle homolog 2
1448661_at	385	(± 42)	189	(± 47)	0.0057	-2.04	Plcb3	phospholipase C, beta 3
1439797_at	306	(± 67)	100	(± 27)	0.0078	-3.05	Ppard	peroxisome proliferator activator receptor delta
1426401_at	1094	(± 85)	525	(± 41)	0.0005	-2.08	Ppp3ca	protein phosphatase 3, catalytic subunit, alpha isoform
1427468_at	1103	(± 170)	207	(± 169)	0.0029	-5.32	Ppp3cb	protein phosphatase 3, catalytic subunit, beta isoform
1450368_a_at	186	(± 53)	83	(± 16)	0.0321	-2.23	Ppp3r1	protein phosphatase 3, regulatory subunit B, alpha isoform (calcineurin B, type I)
1452878_at	366	(± 35)	172	(± 32)	0.0021	-2.13	Prkce	protein kinase C, epsilon
1448695_at	242	(± 84)	70	(± 28)	0.0284	-3.44	Prkci	protein kinase C, iota
1424287_at	190	(± 55)	34	(± 12)	0.0084	-5.53	Prkx	protein kinase, X-linked
1451358_a_at	2310	(± 218)	1154	(± 250)	0.0038	-2.00	Racgap1	Rac GTPase-activating protein 1
1416577_a_at	5908	(± 412)	11936	(± 1884)	0.0056	2.02	Rbx1	ring-box 1
1423444_at	1347	(± 351)	657	(± 105)	0.0311	-2.05	Rock1	Rho-associated coiled-coil containing protein kinase 1
1425465_a_at	611	(± 128)	194	(± 73)	0.0080	-3.15	Senp2	SUMO/sentrin specific peptidase 2
1416594_at	722	(± 105)	300	(± 44)	0.0030	-2.41	Sfrp1	secreted frizzled-related protein 1
1422485_at	2828	(± 354)	1189	(± 425)	0.0068	-2.38	Smad4	MAD homolog 4
1434644_at	780	(± 274)	29	(± 15)	0.0090	-27.22	Tbl1x	transducin (beta)-like 1 X-linked
1429427_s_at	221	(± 5)	460	(± 97)	0.0132	2.08	Tcf7l2	transcription factor 7-like 2, T-cell specific, HMG-box
1455592_at	1336	(± 506)	306	(± 101)	0.0258	-4.37	Vangl2	vang-like 2 (van Gogh homolog)
1448818_at	212	(± 68)	82	(± 16)	0.0326	-2.60	Wnt5a	wingless-related MMTV integration site 5A
1420892_at	645	(± 120)	213	(± 15)	0.0035	-3.03	Wnt7b	wingless-related MMTV integration site 7B

Components of the Wnt pathway were identified according to KEGG and GenMAPP annotation. Affymetrix probe ID numbers are given for representative probe sets. Means of signal intensities and standard deviations were rounded the next full figure; p-values were rounded to the fourth digit after the period except where otherwise indicated; fold-change values were rounded to the second digit after the period. Negative fold-change values indicate reduced expression level in diabetes-exposed embryos.

Central nervous system malformations occur in about 5% of children born to diabetic mothers [35], which represents an up to 15-fold higher risk of over pregnancies unaffected by diabetes. Intriguingly, we found seven genes affected by maternal diabetes that previously have been associated with neural tube defects (NTD): Hif1a, Pdgfra, Twsg1, Adam10, Tgfbr1, Tfrc, and Vcl (for references, see Additional file 4). Thus, de-regulated expression of these genes in diabetes-exposed embryos might predispose embryos to neural tube defects. We also analyzed our dataset from Experiment I for differences between the 5 embryos that were exposed to maternal diabetes, of which two exhibited defective closure of the neural tube. In this comparison, we identified only two genes (Etnk1, Gmfb) that passed the criteria filter of >1.5-fold change and both statistical significance tests. Both genes were detected in the initial diabetes-exposed versus normal comparison; we did not discover any genes that were uniquely altered in NTD embryos, implying that differences between NTD-affected and -unaffected individuals with regard to gene expression are mostly quantitative. Our results are consistent with the hypothesis that diabetes of the mother alters expression of specific known heart-defect and neural-tube-defect genes, and that these genes may be responsible for the birth defects in diabetic pregnancies.

### Confirmation of major findings by a separate profiling experiment

Our intial survey employed embryo samples that came from two pregnant females: one STZ-treated diabetic and a control untreated dam. This presents the theoretical possibility that any differences between progeny of the two dams reflect differences between pregnancies in addition to diabetic state. Also, we used individual embryo samples, and this approach is likely to incur substantial variability in the data and thus understimation of molecular changes. To address both concerns, we conducted Experiment II, in which equal amounts of RNA was pooled from three embryos of same gestational age into one sample, with each embryo derived from a different dam; for the diabetic as well as the control condition, we prepared three such pools, respectively (Figure 1, Panel B). Taking advantage of technical advances, these samples were processed and hybridized to the Affymetrix Mouse 430 2.0 chip, which surveys 39000 transcripts. Using the same analysis criteria as before, we identified 2231 transcripts of which 276 (12.37%) showed increased levels of expression in the diabetes-exposed samples, and 1955 (87.63%) exhibited decreased expression compared to the controls (Figure 1, Panel D). Thus, we confirm the general trend in the results from Experiment I. Of the differentially expressed transcripts, 179 lacked identifying features, such as a name, RefSeq or ENSEMBLE IDs, or Unigene Mm. cluster number; they also lacked any annotation information. This left us with 2052 annotatable genes. Classification by molecular functions revealed a distribution of molecular properties (Figure 3, Panel B) highly similar to that in Experiment I (Figure 3, Panel A). Again, genes encoding transcription factor and DNA-binding regulatory molecules were significantly enriched, accounting for 16.3% of the deregulated genes; strong enrichment was confirmed by DAVID annotation. Annotation for function in vivo identified 1836 gene entries in MGI; for 1095 of those, phenotype information was not available. However, 747 genes were associated with documented phenotypes in mouse mutants, of which 388 are developmental phenotypes by virtue of embryonic, neonatal, or perinatal death of homozygous mutant offspring. Again, the distribution of particular phenotypes in Experiment II (Figure 3, Panel D) was very similar to that of Experiment I (Figure 3, Panel C). Metabolic abnormalities were reported for mutants of 46 genes, and evidence for abnormal growth (pre- and post-natal) was obtained for 279 genes. Most notably, 161 genes are known to be associated with heart defects when mutated, and 112 genes are known to play causal roles in neural tube defects. This is only a fraction (35%) of the more than 300 NTD genes contained in the MGI database (as of October 1, 2008). Similarly, from the published collection of 170 mouse mutants with neural tube defects [37] for which the underlying molecular defect is known, 55 genes (32% of 170) were identified in Experiment II. Taken together, these results indicate that maternal diabetes affects specific pathogenic pathways leading to NTDs. Except for two genes, all NTD genes exhibited decreased expression on the arrays. In summary, the main findings of the initial microarray experiment were confirmed.

Indeed, of the 126 genes whose expression was altered by more than 2-fold in Experiment I, 67 were also recovered above the 2-fold change cut-off in Experiment II. Of the 378 probe sets with expression level altered between 1.5-fold and 2-fold, 187 were shared. Thus, of the 504 probe sets with altered expression in Experiment I, more than half (254) were confirmed in Experiment II, providing independent validation for the major results of the first experiment. This 50% confirmation rate for independent microarray experiments in the same biological paradigm agrees well with similar findings for independent yeast microarray results [38]. Thus, employing individual embryo samples as well as a pooling strategy, we have identified molecular targets in the embryo that respond to maternal diabetes. Also noteworthy is that Experiment II confirmed our earlier candidate gene studies that showed components of the Wnt pathway altered in diabetes-exposed embryos [25]. In fact, 43 genes with roles in Wnt signaling are affected by maternal diabetes (Table 4); with exception of Cank2d, Rbx1 and Tcf7l2 (which are upregulated), the expression levels of all of these genes are decreased in diabetes-exposed embryos compared to controls. This finding provides further support for our hypothesis that maternal diabetes affects specific developmental programs.

Using mRNA from whole individual embryos allowed us to survey a broad range of embryonic tissues that are potentially affected by maternal diabetes. This approach might have "missed" effects on genes that are expressed only in small cell populations of the embryo. However, in Experiment II, we identify 19 of the 47 published genes that were found altered more than 1.5-fold in microarray analysis of cranial neural tube tissue from diabetes-exposed embryos with neural tube defects at E 11.5 [39], and four of those genes are shared with Experiment I. Concordance was found for increased expression of Bnip3, and for decreased levels of En2, Hes6, Ina, Map3k7, Med1, Msx1, Mtap1B, Ngn2, Notch1, TgfβII, Doublecortin, Protocadherin18, Tgfβρεχεπτορ1, TopoIIβ, with the latter four genes confirmed also in Experiment I. Notch3, Nr2f2, Shh, and Tial1, were increased in dissected neural tube [39] but decreased in whole embryos, indicating that they may be deregulated in multiple tissues. Nonetheless, the overlap between results from different laboratories, despite differences in experimental design, provides additional validation to our findings.

We cannot currently distinguish which of the changes in gene expression are in direct response to the diabetic milieu, and which are indirect changes downstream of altered transcription factor expression, potentially increased hypoxia [14] or alterations in yolk sac [40] or placenta [Salbaum, Kruger, Pavlinkova, Zhang, and Kappen, manuscript submitted]. In this regard, it is interesting to note that we find no congruence to genes reported as affected by maternal diabetes in yolk sac of E12 rat embryos [41]. This indicates not only that both yolk sac and embryo gene expression are affected by maternal diabetes, but that extra-embryonic tissues respond differently than the embryo proper. It is noteworthy that among the deregulated genes with known phenotypes in mouse mutants, over 100 have been reported to be associated with placental alterations. Even though we have only surveyed the embryo proper, this is suggestive evidence that placental gene expression may also be altered in diabetic pregnancies. Our findings are consistent with the idea that altered gene expression in the embryo, as de-regulated by maternal diabetes, plays an important role in the pathogenesis of diabetes-induced birth defects [2,42].

### Implications for prevention of adverse outcomes from diabetic pregnancies

High glucose levels during critical periods of morphogenesis appear to be the major teratogen in diabetic pregnancy. In experimental animals, excess glucose is sufficient to cause dysmorphogenesis of embryos in glucose-injected dams or in whole embryo culture [11,43-45]. The precise mechanism(s) by which hyperglycemia induces diabetic embryopathy is(are) not clear, although involvement of the Glut2 (Slc2a2) transporter has been demonstrated [45]. Several studies report increased oxidative stress in embryos in a diabetic environment, and the administration of antioxidants, such as vitamins C or E, can reduce the occurrence of developmental defects [13,17,46,47]. Which genes are functionally involved in these responses in diabetes-exposed embryos, and which mechanisms provide for the protective effect of anti-oxidant treatment in diabetic embryopathy remains to be investigated, but it is likely that one or more of the genes we have identified constitute targets in the antioxidant response. Similarly, folate supplementation has been shown to be protective against NTDs in diabetic pregnancies [46,48]. Interestingly, the gene encoding platelet derived growth factor receptor α (Pdgfrα), mutants of which exhibit neural tube defects [49], is folate-responsive in mice [50]. Genes whose expression is altered in diabetes-exposed embryos thus represent excellent candidates for folate-responsive genes, and may mediate the beneficial effect of folate in the prevention of neural tube and other developmental defects.

## Methods

### Animals

Diabetes was induced in 7–9 week old female FVB mice by two intraperitoneal injections of 100 mg/kg body weight Streptozotocin in 50 mM sodium citrate buffer at pH4.5 (STZ; Sigma, St. Louis, MO) within a one-week interval. The dams were set up for mating no earlier than 7 days after the last injection, and the day of detection of a vaginal plug was counted as day 0.5 of gestation. We used embryos only from dams (n = 11) whose blood glucose levels exceeded 250 mg/dl; average glucose levels were 148 mg/dl(± 18) before STZ treatment, 337 mg/dl(± 79) on the day of mating, and 528 mg/dl(± 70) on the day of embryo harvest.

### Microarray Analysis

Total RNA was isolated from embryos at embryonic day 10.5 (E10.5) using Trizol^® ^(Invitrogen, Carlsbad, CA). We processed 2 controls and five diabetes-exposed embryos; the latter group included two specimen with neural tube defects (NTD) so as to capture the full phenotype spectrum of diabetes-exposure in pregnancy. Individual RNA samples (5 μg) from whole embryos were reverse transcribed (Invitrogen) and labeled (Affymetrix, Santa Clara, CA). In Experiment I, samples were individually hybridized to 7 Affymetrix430A2.0 chips, which were scanned using a GeneChip3000 scanner; Affymetrix GCOS imaging software was used for quality control. In Experiment II, equal amounts of RNA prepared from 3 individual embryos were pooled into one sample; each embryo was from a different pregnancy and three such pools were constructed for a total of 9 control embryos, and independently, for 9 diabetes-exposed embryos; all embryos were morphologically normal. Expression levels and "Present", "Marginal", "Absent" flags were determined with default parameters through comparison of matched and mismatched oligonucleotides for the respective gene sequence.

Statistical analyses were performed using GeneSpring7 (Silicon Genetics, Redwood City, CA) and CyberT [51]. We grouped the data for control embryos and those for diabetes-exposed embryosm respectively, and filtered results in three steps: (i) "expression", i.e. "present" or "marginal" in at least one of seven samples; (ii) "statistical significance" between control and experimental samples of P < 0.05 in both CyberT and the t-test in GeneSpring; and (iii) "fold change", i.e. difference between control and diabetes-exposed samples of beyond either two-fold or 1.5-fold. The rationale for employing complementary data analysis packages and details of data transformation have been described elsewhere [52].

Of 22690 probe sets present on the arrays , 15364 probes exhibited signals in at least one of the 7 samples, and 302 probe sets differed by more than two fold between these samples. Of these, 180 probe sets passed the t-test in GeneSpring (P < 0.05; Welch's test assuming unequal variances; false discovery rate set at 0.05), and 174 probes yielded P-values below P < 0.05 in Cyber-T. Permutation of the order of tests (significance first, fold-change second) identified differential signals from 2262 probe sets (Cyber-T), with 575 probes displaying differences between 1.5 and 2-fold, and 174 probes with differences greater than 2-fold between controls and exposed embryos. Regardless of order of filtering criteria, identical sets of probes were recovered, thus validating the analysis process. Between Cyber-T and GeneSpring, 145 sets passed both statistical filters, and after removal of duplicates, 126 genes were found to be differentially expressed above the 2-fold cut-off criterion.

For the second experiment, Affymetrix Mouse 430 2.0 arrays were used, which contain 45101 probe sets . 29687 probe sets exhibited signals in at least one of 6 samples; differences reached statistical significance at p < 0.05 for 9835 probes in the t-test (P < 0.05; Welch's test assuming unequal variances; false discovery rate set at 0.05) implemented in GeneSpring (GX version 9). 5915 probe sets exhibited differences greater than 1.5-fold, with 2796 differentially expressed greater than 2-fold. Cyber-T identified 13770 probe sets with statistical significance, of which 3992 exceeded the 1.5-fold change level, and an additional 4601 exceeded the greater than 2-fold criterion. After removal of internal controls, 5688 probe sets passed the filtering criteria for statistical significance in both Cyber-T and GeneSpring and exhibited >1.5-fold change between experimental and control samples, of which 2634 probe sets were identified with greater than 2-fold differential expression. Reduction of duplicates for a given gene was done by judgement call factoring in signal intensity, P-value, distribution of calls ("Present" was judged as more reliable than "Marginal") and fold-change; only one entry per gene was retained for a total of 2231 transcripts with differential expression greater than 2-fold.

The primary data files are available at the NCBI Gene Expression Omnibus repository (Accession number GSE9675).

### Quantitative Real-time PCR

Quantitative Real-Time PCR (Q-RT-PCR) using an ABI Prism7000 instrument was performed as described [53] on cDNA samples from individual diabetes-exposed embryos (5 litters) and controls (4 litters), or pools of 4–5 control embryos from the same litter (4 litters) isolated at E10.5 (for details, see legend to Table 2). At E9.5, 6 control and 9 diabetes-exposed embryos were selected from 3 litters each, respectively, and E8.5 embryos were from 4 litters (10 controls) and 5 litters (9 diabetes-exposed embryos). All embryos used for Q-RT-PCR were morphologically normal. Normalization was done to Polymerase epsilon 4 (Pole4) cDNA in the same sample; Pole4 levels were unaffected by maternal diabetes on Experiment I and Experiment II arrays. Differences between samples (n = individual embryos except where noted otherwise) were evaluated for statistical significance using an unpaired two-tailed t-test. Primers (Additional file 5) were positioned across exon-exon junctions to exclude amplification of potentially contaminating DNA. The amplification products were designed to originate from a different region of the mRNA than that detected by probes on the microarray, in order to provide independent confirmation of expression measurements.

### Annotation for tissue expression, function and mutant mouse phenotypes

Information on gene expression in embryos, where available, was collected from MGI . Molecular function attributes were based on GO-annotation (NetAffx™ , updated as of July 21, 2008), supplemented with information from ENSEMBL and UCSC genome browsers and PubMed. Information on mutant phenotypes was obtained from MGI (as of October 21, 2008) for null and conditional alleles.

### Transcription factor binding site prediction

Whole Genome rVISTA  was used to identify transcription factor binding sites that are conserved between mouse and human and are over-represented in the 5 Kb upstream regions of our maternal diabetes affected genes relative to all 5 Kb upstream regions in the human genome (P-value < 0.006).

## Authors' contributions

GP performed microarray analyses and PCR assays, collected information for annotations and drafted a first version of the manuscript, JMS oversaw the statistical analysis by Cyber-T, designed experiment II and led the annotation effort, CK conceived of the study, performed the annotation for experiment II and wrote the manuscript.

## Supplementary Material

Additional file 1 **Genes with altered expression in diabetes-exposed embryos**. The file contains a list of genes identified by microarray analysis. Click here for file

Additional file 2 **Transcription factor binding sites overrepresented in promoters of genes affected by maternal diabetes**. The file contains a list of putative transcription factor binding sites and respective references. Click here for file

Additional file 3 **Known HIF1 target genes that exhibit altered expression in diabetes-exposed embryos**. The file contains a list of HIF1 target genes and respective references. Click here for file

Additional file 4 **References for in vivo function of genes affected in diabetes exposed embryos**. The file contains a list of references for the in vivo function of particular genes. Click here for file

Additional file 5 **Primer sequences for quantitative RT-PCR**. The file contains a list of RefSeqIDs, position information and sequences for primers to amplify particular genes. The amplification rate for each primer pair is also listed. Click here for file
