# Supplementary material for: Maternal diabetes alters transcriptional programs in the developing embryo
Source: BMC Genomics. 2009 Jun 18;10:274. doi: 10.1186/1471-2164-10-274 (PMC2715936; doi:10.1186/1471-2164-10-274)
Supplement: Additional file 1 — Genes with altered expression in diabetes-exposed embryos. The file contains a list of genes identified by microarray analysis. [file 1471-2164-10-274-S1.doc]

Additional file 1: **Genes with altered expression in diabetes-exposed embryos**

| Gene symbol | Affymetrix | RefSeq | | CyberT | | | Fold | | Gene Name | |
| --- | --- | --- | --- | --- | --- | --- | --- | --- | --- | --- |
|  | probe ID | Transcript ID | | (P value) | | | change | |  | |
| Genes with decreased expression | | | | |  | | |  | |  |
| 1300007C21Rik | 1431213_a_at | NM_001012326 | 0.0018 | | | -2.70 | | | RIKEN cDNA 1300007C21 gene | |
| 2610020O08Rik | 1419763_at | NM_025937 | 0.0120 | | | -2.17 | | | nuclear NF-kappaB activating protein | |
| 6330503C03Rik | 1455421_x_at | NM_029528 | 0.0173 | | | -2.02 | | | RIKEN cDNA 6330503C03 gene | |
| 6330527O06Rik | 1423853_at | NM_029530 | 0.0105 | | | -2.22 | | | RIKEN cDNA 6330527O06 gene | |
| 6330578E17Rik | 1426356_at | NM_198006 | 0.0290 | | | -2.86 | | | RIKEN cDNA 6330578E17 gene | |
| Abcb7 | 1427490_at | XM_907304 | 0.0001 | | | -3.17 | | | ATP-binding cassette, sub-family B (MDR/TAP) member 7 | |
| Adam10 | 1450104_at | NM_007399 | 0.0024 | | | -2.37 | | | a disintegrin and metallopeptidase domain 10 | |
| Agtr2 | 1415832_at | NM_007429 | 0.0193 | | | -2.43 | | | angiotensin II receptor type 2 | |
| Ap1g1 | 1423388_at | NM_009677 | 0.0005 | | | -2.18 | | | adaptor protein complex AP-1 gamma 1 subunit | |
| Api5 | 1439214_a_at | NM_007466 | 0.0004 | | | -2.30 | | | apoptosis inhibitor 5 | |
| Aplp2 | 1432344_a_at | NM_009691 | 0.0052 | | | -2.20 | | | amyloid beta (A4) precursor-like protein 2 | |
| Aqr | 1430971_a_at | NM_009702 | 0.0025 | | | -2.02 | | | aquarius | |
| Arid4b | 1460384_a_at | NM_198122 | 0.0017 | | | -2.24 | | | AT rich interactive domain 4B (Rbp1 like) | |
| Arih2 | 1418523_at | NM_011790 | 0.0372 | | | -2.09 | | | ariadne homolog 2 (Drosophila) | |
| Arl5a | 1447926_at | NM_182994 | 0.0007 | | | -2.33 | | | ADP-ribosylation factor-like 5A | |
| Atrx ^a^ | 1420946_at | NM_009530 | 0.0016 | | | -2.15 | | | alpha thalassemia/mental retardation syndrome X-linked homolog (human) | |
| Baz1b | 1450068_at | NM_011714 | 0.0012 | | | -2.38 | | | bromodomain adjacent to zinc finger domain 1B | |
| Bcl11a | 1426552_a_at | NM_016707 | 0.0029 | | | -2.35 | | | B-cell CLL/lymphoma 11A (zinc finger protein) | |
| Birc4 | 1456088_at | NM_009688 | 0.0006 | | | -2.54 | | | baculoviral IAP repeat-containing 4 | |
| Cacna2d1 | 1425861_x_at | NM_009784 | 0.0098 | | | -2.73 | | | calcium channel, voltage-dependent alpha2/delta subunit 1 | |
| Creb1 | 1452529_a_at | NM_133828 | 0.0007 | | | -3.33 | | | cAMP responsive element binding protein 1 | |
| Crsp2 | 1450229_at | NM_012005 | 0.0180 | | | -2.27 | | | cofactor required for Sp1 transcriptional activation subunit 2 | |
| Ctse | 1427797_s_at | NM_007799 | 0.0167 | | | -2.14 | | | cathepsin E | |
| Cxadr | 1452391_at | NM_009988 | 0.0034 | | | -2.00 | | | coxsackievirus and adenovirus receptor | |
| Dcp1a | 1431933_a_at | NM_133761 | 0.0335 | | | -2.81 | | | decapping enzyme | |
| Dcx ^a^ | 1418141_at | NM_010025 | 0.0001 | | | -3.05 | | | doublecortin | |
| Ddx3y ^b^ | 1452077_at | NM_012008 | 0.0062 | | | -6.23 | | | DEAD (Asp-Glu-Ala-Asp) box polypeptide 3, Y-linked | |
| Dnm1l | 1452638_s_at | NM_152816 | 0.0039 | | | -2.90 | | | dynamin 1-like | |
| Dysf | 1436261_at | NM_021469 | 0.0277 | | | -2.68 | | | Dysferlin | |
| Efnb2 | 1449549_at | NM_010111 | 0.0437 | | | -2.04 | | | ephrin B2 | |
| Eif3s10 | 1416661_at | NM_010123 | 0.0366 | | | -2.19 | | | eukaryotic translation initiation factor 3 subunit 10 (theta) | |
| Epb4.1l2 | 1433492_at | NM_013511 | 0.0055 | | | -2.09 | | | erythrocyte protein band 4.1-like 2 | |
| Epha3 | 1426057_a_at | NM_010140 | 0.0059 | | | -2.56 | | | Eph receptor A3 | |
| Etnk1 ^a^ | 1454633_at | XM_908334 | 0.0008 | | | -2.40 | | | ethanolamine kinase 1 | |
| Exod1 | 1454229_a_at | NM_027698 | 0.0189 | | | -4.28 | | | exonuclease domain containing 1 | |
| Gad1 | 1416561_at | NM_008077 | 0.0082 | | | -2.28 | | | glutamic acid decarboxylase 1 | |
| Ghr | 1451871_a_at | NM_010284 | 0.0015 | | | -3.34 | | | growth hormone receptor | |
| Gmfb ^a^ | 1431686_a_at | NM_022023 | 0.0008 | | | -3.21 | | | glia maturation factor beta | |
| Gopc | 1450153_at | NM_053187 | 0.0163 | | | -2.31 | | | golgi associated PDZ and coiled-coil motif containing | |
| Gpr65 | 1449175_at | NM_008152 | 0.0065 | | | -2.85 | | | G-protein coupled receptor 65 | |
| Grb10 ^a^ | 1425457_a_at | NM_010345 | 0.0072 | | | -2.72 | | | growth factor receptor bound protein 10 | |
| Hdlbp ^a^ | 1449615_s_at | NM_133808 | 0.0025 | | | -2.30 | | | high density lipoprotein (HDL) binding protein | |
| Heatr1 | 1452419_at | NM_144835 | 0.0065 | | | -2.17 | | | HEAT repeat containing 1 | |
| Hif1a | 1448183_a_at | NM_010431 | 0.0041 | | | -2.52 | | | hypoxia inducible factor 1 alpha subunit | |
| Hist1h2bc | 1452540_a_at | NM_023422 | 0.0009 | | | -2.22 | | | histone 1 H2bc | |
| Hs6st2 | 1420938_at | NM_015819 | 0.0003 | | | -2.98 | | | heparan sulfate 6-O-sulfotransferase 2 | |
| Il6st | 1421239_at | NM_010560 | 0.0198 | | | -2.28 | | | interleukin 6 signal transducer | |
| Itgav | 1421198_at | NM_008402 | 0.0006 | | | -3.46 | | | integrin alpha V | |
| Ivns1abp | 1420961_a_at | NM_054102 | 0.0191 | | | -2.04 | | | influenza virus NS1A binding protein | |
| Jarid1d ^a,b^ | 1424903_at | NM_011419 | 0.0281 | | | -4.63 | | | jumonji AT rich interactive domain 1D (Rbp2 like) | |
| Kif11 | 1452315_at | NM_010615 | 0.0001 | | | -2.22 | | | kinesin family member 11 | |
| Kras | 1426229_s_at | NM_021284 | 0.0453 | | | -2.02 | | | v-Ki-ras2 Kirsten rat sarcoma viral oncogene homolog | |
| Lin7c | 1449262_s_at | NM_011699 | 0.0005 | | | -2.44 | | | lin-7 homolog C (C. elegans) | |
| LOC640370 | 1432646_a_at | XM_001002907 | 0.0238 | | | -2.83 | | | hypothetical LOC640370 | |
| LOC669660 | 1422862_at | XM_976375 | 0.0001 | | | -2.08 | | | similar to PDZ and LIM domain protein 5 (Enigma homolog) | |
| Mapk10 | 1437195_x_at | NM_009158 | 0.0033 | | | -5.06 | | | mitogen activated protein kinase 10 | |
| Mbtps1 | 1431385_a_at | NM_019709 | 0.0160 | | | -2.58 | | | membrane-bound transcription factor peptidase site 1 | |
| Msl31 | 1448645_at | NM_010832 | 0.0365 | | | -2.08 | | | male-specific lethal-3 homolog 1 (Drosophila) | |
| Mtap2 | 1421328_at | NM_008632 | 0.0000 | | | -3.02 | | | microtubule-associated protein 2 | |
| Ndst1 | 1422044_at | NM_008306 | 0.0133 | | | -3.38 | | | N-deacetylase/N-sulfotransferase (heparan glucosaminyl) 1 | |
| Nedd4 | 1421955_a_at | NM_010890 | 0.0031 | | | -2.02 | | | neural precursor cell expressed developmentally down-regulated gene 4 | |
| Nsd1 | 1420881_at | NM_008739 | 0.0008 | | | -2.63 | | | nuclear receptor-binding SET-domain protein 1 | |
| Pcdh18 | 1430427_a_at | NM_130448 | 0.0017 | | | -5.77 | | | protocadherin 18 | |
| Pdgfra | 1421916_at | NM_011058 | 0.0010 | | | -2.01 | | | platelet derived growth factor receptor alpha polypeptide | |
| Pelp1 | 1449973_a_at | NM_029231 | 0.0296 | | | -2.13 | | | proline, glutamic acid and leucine rich protein 1 | |
| Phip ^a^ | 1451805_at | XM_001004343 | 0.0006 | | | -2.76 | | | pleckstrin homology domain interacting protein | |
| Pik3c2a | 1425862_a_at | NM_011083 | 0.0022 | | | -2.31 | | | phosphatidylinositol 3-kinase C2 domain containing alpha polypeptide | |
| Pkia ^a^ | 1420859_at | NM_008862 | 0.0216 | | | -2.18 | | | protein kinase inhibitor, alpha | |
| Plekha5 | 1425544_at | NM_144920 | 0.0190 | | | -2.75 | | | pleckstrin homology domain containing family A member 5 | |
| Ptprk | 1431680_a_at | NM_008983 | 0.0125 | | | -2.47 | | | protein tyrosine phosphatase receptor type K | |
| Ptprs | 1426795_at | NM_011218 | 0.0344 | | | -2.25 | | | protein tyrosine phosphatase receptor type S | |
| Pxn | 1426085_a_at | NM_011223 | 0.0035 | | | -2.14 | | | paxillin | |
| Rabgap1 | 1460486_at | NM_001033960 | 0.0438 | | | -2.76 | | | RAB GTPase activating protein 1 | |
| Rb1cc1 | 1449292_at | NM_009826 | 0.0363 | | | -2.14 | | | RB1-inducible coiled-coil 1 | |
| Rnf14 | 1431030_a_at | NM_020012 | 0.0006 | | | -3.48 | | | ring finger protein 14 | |
| Rnpc2 | 1438397_a_at | NM_133242 | 0.0140 | | | -2.20 | | | RNA-binding region (RNP1, RRM) containing 2 | |
| Rod1 | 1424083_at | NM_144904 | 0.0004 | | | -2.32 | | | ROD1 regulator of differentiation 1 (S. pombe) | |
| Rp2h | 1419585_at | NM_133669 | 0.0002 | | | -2.06 | | | retinitis pigmentosa 2 homolog (human) | |
| Scd2 | 1415824_at | NM_009128 | 0.0107 | | | -2.64 | | | stearoyl-Coenzyme A desaturase 2 | |
| Sel1h | 1430692_a_at | NM_011344 | 0.0004 | | | -2.34 | | | Sel1 (suppressor of lin-12) 1 homolog (C. elegans) | |
| Sema3a | 1449865_at | NM_009152 | 0.0003 | | | -2.05 | | | sema domain (semaphorin) 3A * | |
| Setdb1 | 1416670_at | NM_018877 | 0.0051 | | | -2.32 | | | SET domain bifurcated 1 | |
| Sgpl1 | 1415893_at | NM_009163 | 0.0210 | | | -2.03 | | | sphingosine phosphate lyase 1 | |
| Sncg | 1417788_at | NM_011430 | 0.0073 | | | -2.34 | | | synuclein, gamma | |
| Sptlc1 | 1436727_x_at | NM_009269 | 0.0139 | | | -2.39 | | | serine palmitoyltransferase, long chain base subunit 1 | |
| Stam2 | 1416975_at | NM_019667 | 0.0479 | | | -2.40 | | | signal transducing adaptor molecule (SH3 domain and ITAM motif) 2 | |
| Stom | 1419099_x_at | NM_013515 | 0.0169 | | | -2.27 | | | stomatin | |
| Stx17 | 1427924_at | NM_026343 | 0.0321 | | | -2.31 | | | syntaxin 17 | |
| Supt16h | 1449578_at | NM_033618 | 0.0161 | | | -2.02 | | | suppressor of Ty 16 homolog (S. cerevisiae) | |
| Syncrip ^a^ | 1422769_at | NM_019796 | 0.0040 | | | -2.04 | | | synaptotagmin binding, cytoplasmic RNA interacting protein | |
| Tfrc | 1422967_a_at | NM_011638 | 0.0023 | | | -2.20 | | | transferrin receptor | |
| Tgfbr1 ^a^ | 1420893_a_at | NM_009370 | 0.0002 | | | -3.26 | | | transforming growth factor beta receptor I | |
| Tm9sf3 | 1448375_at | NM_133352 | 0.0012 | | | -2.36 | | | transmembrane 9 superfamily member 3 | |
| Tnks2 | 1452772_at | XM_001000101 | 0.0005 | | | -2.82 | | | tankyrase, TRF1-interacting ankyrin-related ADP-ribose polymerase 2 | |
| Top2b | 1448458_at | NM_009409 | 0.0034 | | | -4.09 | | | topoisomerase (DNA) II beta | |
| Trim2 | 1417029_a_at | NM_030706 | 0.0039 | | | -2.12 | | | tripartite motif protein 2 | |
| Trim44 | 1421869_at | NM_020267 | 0.0039 | | | -2.42 | | | tripartite motif-containing 44 | |
| Tubb2b | 1449682_s_at | NM_023716 | 0.0351 | | | -2.08 | | | tubulin beta 2b | |
| Twsg1 | 1426179_a_at | NM_023053 | 0.0002 | | | -4.91 | | | twisted gastrulation homolog 1 (Drosophila) | |
| Usp12 | 1425805_a_at | NM_011669 | 0.0004 | | | -2.71 | | | ubiquitin specific peptidase 12 | |
| Uty ^a,b^ | 1422247_a_at | NM_009484 | 0.0204 | | | -3.34 | | | ubiquitously transcribed tetratricopeptide repeat gene, Y chromosome | |
| Vcl | 1416157_at | NM_009502 | 0.0020 | | | -2.37 | | | vinculin | |
| Vps35 | 1415784_at | NM_022997 | 0.0012 | | | -2.06 | | | vacuolar protein sorting 35 | |
| Ywhag | 1420816_at | NM_018871 | 0.0021 | | | -2.73 | | | 3-monooxygenase/tryptophan 5-monooxygenase activation protein | |
| Zcsl3 | 1451389_at | NM_026992 | 0.0078 | | | -2.52 | | | zinc finger CSL-type containing 3 | |
| Zfa | 1422249_s_at | NM_009540 | 0.0001 | | | -7.58 | | | zinc finger protein autosomal | |
| Zfp294 | 1427950_at | XM_128374 | 0.0105 | | | -2.25 | | | zinc finger protein 294 | |
| Zfp60 | 1422954_at | NM_029531 | 0.0213 | | | -2.03 | | | zinc finger protein 60 | |
| Genes with increased expression | | |  | | |  | | |  | |
| Aldh18a1 | 1437620_x_at | NM_019698 | 0.0315 | | | 2.09 | | | Aldehyde dehydrogenase 18 family member A1 | |
| BC067396 | 1424152_at |  | 0.0006 | | | 2.30 | | | CDNA clone IMAGE:30031514 | |
| Blvrb | 1451386_at | NM_144923 | 0.0261 | | | 2.12 | | | biliverdin reductase B (flavin reductase (NADPH)) | |
| Cited4 | 1425400_a_at | NM_019563 | 0.0026 | | | 2.11 | | | Cbp/p300-interacting transactivator with Glu/Asp carboxy-terminal domain 4 | |
| Gmpr | 1448530_at | NM_025508 | 0.0182 | | | 2.01 | | | guanosine monophosphate reductase | |
| Hmga1 | 1416184_s_at | NM_001025427 | 0.0060 | | | 2.13 | | | high mobility group AT-hook 1 | |
| Klf9 | 1456341_a_at | NM_010638 | 0.0170 | | | 5.00 | | | Kruppel-like factor 9 | |
| Lin28 | 1437752_at | NM_145833 | 0.0182 | | | 2.00 | | | lin-28 homolog (C. elegans) | |
| Mt2 | 1428942_at | NM_008630 | 0.0053 | | | 3.45 | | | metallothionein 2 | |
| Ogt | 1436780_at | NM_139144 | 0.0310 | | | 2.52 | | | O-linked N-acetylglucosamine (GlcNAc) transferase | |
| Pabpc1 | 1453840_at | NM_008774 | 0.0402 | | | 2.13 | | | poly A binding protein cytoplasmic 1 | |
| Pfkl | 1439148_a_at | NM_008826 | 0.0052 | | | 2.02 | | | phosphofructokinase liver, B-type | |
| Ptp4a3 | 1418181_at | NM_008975 | 0.0076 | | | 2.20 | | | protein tyrosine phosphatase 4a3 | |
| Sfrs2 | 1427816_at | NM_011358 | 0.0311 | | | 3.92 | | | splicing factor arginine/serine-rich 2 (SC-35) | |
| Slc16a3 | 1449005_at | NM_030696 | 0.0449 | | | 3.06 | | | solute carrier family 16 (monocarboxylic acid transporters), member 3 | |
| Slc25a22 | 1452653_at | NM_026646 | 0.0362 | | | 2.19 | | | solute carrier family 25 (mitochondrial carrier, glutamate), member 22 | |
| Slc2a1 ^a^ | 1426600_at | NM_011400 | 0.0252 | | | 2.15 | | | solute carrier family 2 (facilitated glucose transporter), member 1 | |
| Tubb2c | 1439416_x_at | NM_146116 | 0.0430 | | | 3.14 | | | tubulin, beta 2c | |
| Upp1 | 1448562_at | NM_009477 | 0.0401 | | | 2.75 | | | uridine phosphorylase 1 | |
| Usp7 | 1419921_s_at | NM_001003918 | 0.0296 | | | 2.49 | | | Ubiquitin specific peptidase 7 | |
| Zfp385 | 1418865_at | NM_013866 | 0.0029 | | | 2.11 | | | zinc finger protein 385 | |
| ^a^ genes detected with two probe sets at significantly altered expression level  ^b^ genes that map to the Y chromosome | | | | | | | | | | |
