# Supplementary material for: Maternal diabetes alters transcriptional programs in the developing embryo
Source: BMC Genomics. 2009 Jun 18;10:274. doi: 10.1186/1471-2164-10-274 (PMC2715936; doi:10.1186/1471-2164-10-274)
Supplement: Additional file 2 — Transcription factor binding sites overrepresented in promoters of genes affected by maternal diabetes. The file contains a list of putative transcription factor binding sites and respective references. [file 1471-2164-10-274-S2.doc]

Additional file 2: **Transcription factor binding sites overrepresented in promoters of genes affected by maternal diabetes.**

| Transcription factor binding sites | Number of genes | References |
| --- | --- | --- |
| *FOXO1/FOXO4 (Forkhead 1/4) | 73/74 | 1, 2 |
| *NRF2 (NF-E2 p45 related factor 2) | 76 | 3, 4 |
|  |  |  |
| HIF1 (Hypoxia inducible factor 1) | 22 | 5 |
| ATF3 (activating transcription factor 3) | 53 | 6 |
| ATF4 (activating transcription factor 4) | 71 | 7 |
| E2F1 (E2F transcription factor 1) | 75 | 8 |
| E2F4 (E2F transcription factor 4) | 33 | 8 |
| EGR1 (Early growth response 1) | 18 | 9 |
| ETS1 (E26 avian leukemia oncogene 1, 5' domain) | 67 | 10 |
| IRF1 (interferon regulatory factor 1) | 62 | 11 |
| NFkappaB (nuclear factor-kappa B) | 52 | 12-14 |
| SOX9 (SRY-box containing gene 9) | 65 | 15 |
| SP3 (trans-acting transcription factor 3) | 53 | 16 |
| XBP1 (X-box binding protein 1) | 18 | 17 |

Legend to Additional file 2:

Motifs were predicted in 5 kilobase upstream regions.

*: Transcription factor involved in oxidative stress responses; all others reported in hypoxia responses.

References:

1. C. Urbich, A. Knau, S. Fichtlscherer, D.H. Walter, T. Bruhl, M. Potente, W.K. Hofmann, S. de Vos, A.M. Zeiher, and S. Dimmeler. 2005. FOXO-dependent expression of the proapoptotic protein Bim: pivotal role for apoptosis signaling in endothelial progenitor cells. Faseb J. 19(8): 974-6.

2. M.A. Essers, L.M. de Vries-Smits, N. Barker, P.E. Polderman, B.M. Burgering, and H.C. Korswagen. 2005. Functional interaction between beta-catenin and FOXO in oxidative stress signaling. Science. 308(5725): 1181-4.

3. L. Leung, M. Kwong, S. Hou, C. Lee, and J.Y. Chan. 2003. Deficiency of the Nrf1 and Nrf2 transcription factors results in early embryonic lethality and severe oxidative stress. J Biol Chem. 278(48): 48021-9.

4. M.J. Calkins, R.J. Jakel, D.A. Johnson, K. Chan, Y.W. Kan, and J.A. Johnson. 2005. Protection from mitochondrial complex II inhibition in vitro and in vivo by Nrf2-mediated transcription. Proc Natl Acad Sci U S A. 102(1): 244-9.

5. N.V. Iyer, L.E. Kotch, F. Agani, S.W. Leung, E. Laughner, R.H. Wenger, M. Gassmann, J.D. Gearhart, A.M. Lawler, A.Y. Yu, and G.L. Semenza. 1998. Cellular and developmental control of O2 homeostasis by hypoxia-inducible factor 1 alpha. Genes Dev. 12(2): 149-62.

6. M. Olbryt, M. Jarzab, J. Jazowiecka-Rakus, K. Simek, S. Szala, and A. Sochanik. 2006. Gene expression profile of B 16(F10) murine melanoma cells exposed to hypoxic conditions in vitro. Gene Expr. 13(3): 191-203.

7. J.D. Blais, V. Filipenko, M. Bi, H.P. Harding, D. Ron, C. Koumenis, B.G. Wouters, and J.C. Bell. 2004. Activating transcription factor 4 is translationally regulated by hypoxic stress. Mol Cell Biol. 24(17): 7469-82.

8. R.S. Bindra, S.L. Gibson, A. Meng, U. Westermark, M. Jasin, A.J. Pierce, R.G. Bristow, M.K. Classon, and P.M. Glazer. 2005. Hypoxia-induced down-regulation of BRCA1 expression by E2Fs. Cancer Res. 65(24): 11597-604.

9. H. Nishi, K.H. Nishi, and A.C. Johnson. 2002. Early Growth Response-1 gene mediates up-regulation of epidermal growth factor receptor expression during hypoxia. Cancer Res. 62(3): 827-34.

10. M. Oikawa, M. Abe, H. Kurosawa, W. Hida, K. Shirato, and Y. Sato. 2001. Hypoxia induces transcription factor ETS-1 via the activity of hypoxia-inducible factor-1. Biochem Biophys Res Commun. 289(1): 39-43.

11. D.S. Tendler, C. Bao, T. Wang, E.L. Huang, E.A. Ratovitski, D.A. Pardoll, and C.J. Lowenstein. 2001. Intersection of interferon and hypoxia signal transduction pathways in nitric oxide-induced tumor apoptosis. Cancer Res. 61(9): 3682-8.

12. E.P. Cummins and C.T. Taylor. 2005. Hypoxia-responsive transcription factors. Pflugers Arch. 450(6): 363-71.

13. C. Michiels, E. Minet, D. Mottet, and M. Raes. 2002. Regulation of gene expression by oxygen: NF-kappaB and HIF-1, two extremes. Free Radic Biol Med. 33(9): 1231-42.

14. R.A. Rupec and P.A. Baeuerle. 1995. The genomic response of tumor cells to hypoxia and reoxygenation. Differential activation of transcription factors AP-1 and NF-kappa B. Eur J Biochem. 234(2): 632-40.

15. J.C. Robins, N. Akeno, A. Mukherjee, R.R. Dalal, B.J. Aronow, P. Koopman, and T.L. Clemens. 2005. Hypoxia induces chondrocyte-specific gene expression in mesenchymal cells in association with transcriptional activation of Sox9. Bone. 37(3): 313-22.

16. D.J. Discher, N.H. Bishopric, X. Wu, C.A. Peterson, and K.A. Webster. 1998. Hypoxia regulates beta-enolase and pyruvate kinase-M promoters by modulating Sp1/Sp3 binding to a conserved GC element. J Biol Chem. 273(40): 26087-93.

17. L. Romero-Ramirez, H. Cao, D. Nelson, E. Hammond, A.H. Lee, H. Yoshida, K. Mori, L.H. Glimcher, N.C. Denko, A.J. Giaccia, Q.T. Le, and A.C. Koong. 2004. XBP1 is essential for survival under hypoxic conditions and is required for tumor growth. Cancer Res. 64(17): 5943-7.
