# Supplementary material for: Maternal diabetes alters transcriptional programs in the developing embryo
Source: BMC Genomics. 2009 Jun 18;10:274. doi: 10.1186/1471-2164-10-274 (PMC2715936; doi:10.1186/1471-2164-10-274)
Supplement: Additional file 3 — Known HIF1 target genes that exhibit altered expression in diabetes-exposed embryos. The file contains a list of HIF1 target genes and respective references. [file 1471-2164-10-274-S3.doc]

Additional file 3: **Known HIF1 target genes that exhibit altered expression in diabetes-exposed embryos.**

|  | Fold change | GeneSpring t-test | CyberT t-test |  |  |  |
| --- | --- | --- | --- | --- | --- | --- |
| Gene name |  |  |  | Phenotype in Knockout | Full gene name | |
| Ets1 | -1.7 | 0.016 | 0.039 | altered B- and T-cell differentiation, premature death ^1-7^ | E26 avian leukemia oncogene 1 5' domain | |
| Trfc | -2.2 | 0.002 | 0.002 | embryonic lethality by E12.5 ^8^ | Transferrin receptor | |
| Aldoa | 1.9 | 0.013 | 0.043 |  | Aldolase 1 A isoform | |
| Cdkn1a | 1.7 | 0.023 | 0.004 | altered T-cell differentiation (Lupus-like), premature death ^9-12^ | Cyclin-dependent kinase inhibitor 1A (P21) | |
| Cited2 | 1.8 | 0.002 | 0.023 | embryonic lethality, heart defects ^13, 14^ | Cbp/p300-interacting transactivator 2 | |
| Glut1 (Slc2a1) | 2.2 | 0.025 | 0.001 | developmental delay by E10.5, lethality by E14 ^15^ | Solute carrier family 2 member 1 | |
| Gpi1 | 1.5 | 0.001 | 0.017 | early embryonic lethality E7.5-8.5 ^16, 17^ | Glucose phosphate isomerase 1 | |
| Pfkl | 2.0 | 0.005 | 0.028 |  | Phosphofructokinase liver B-type | |
| Tpi | 1.6 | 0.001 | 0.037 | embryonic lethality, ENU mutations ^18^ | Triosephosphate isomerase | |
| Bnip3 | 2.8 | 0.005 | NS |  | Bcl2/adenovirus E1B 19kDa-interacting protein 1 NIP3 | |
| Cd99 | 1.6 | 0.028 | NS |  | CD99 antigen | |
| Ddit4 | 1.5 | 0.011 | NS | decreased oxygen-induced retinopathy ^19^ | DNA-damage-inducible transcript 4=RTP801 | |
| Glut3 (Slc2a3) | 2.5 | 0.004 | NS | blastocyst death in antisense experiment ^20^ | Solute carrier family 2 member 3 | |
| Hk2 | 1.6 | 0.042 | NS | embryonic lethality E7.5 ^21^ | Hexokinase 2 | |
| Krt1-18 | 1.8 | 0.025 | NS | viable, liver pathology with age ^22^ | Keratin complex 1 acidic gene 18 | |
| Krt1-19 | 1.9 | 0.008 | NS | viable, double KO (Krt18+19) is embryonic lethal by E9.5 ^23^ | Keratin complex 1 acidic gene 19 | |
| Ldha | 1.5 | 0.006 | NS | ENU mutation; embryonic lethality ^24, 25^ | Lactate dehydrogenase 1, A chain | |
| P4ha1 | 1.8 | 0.028 | NS |  | Procollagen-proline 2-oxoglutarate 4-dioxygenase | |
| Pfkfb3 | 1.5 | 0.005 | NS | embryonic lethality E8 ^26^ | 6-phosphofructo-2-kinase/fructose-2,6-biphosphatase 3 | |
| Tgm2 | 1.6 | 0.01 | NS | impaired glucose-stimulated insulin secretion ^27^ | Transglutaminase 2 C polypeptide | |
| Vegfa | 1.7 | 0.015 | NS | embryonic lethality E8.5 ^28^ | Vascular endothelial growth factor A | |
| Vegfb | 1.9 | NS | 0.01 | coronary vasculature, heart function ^29, 30^ | Vascular endothelial growth factor B | |

NS = not significant at P<0.05

References:

1. D. Wang, S.A. John, J.L. Clements, D.H. Percy, K.P. Barton, and L.A. Garrett-Sinha. 2005. Ets-1 deficiency leads to altered B cell differentiation, hyperresponsiveness to TLR9 and autoimmune disease. Int Immunol. 17(9): 1179-91.

2. J.L. Clements, S.A. John, and L.A. Garrett-Sinha. 2006. Impaired generation of CD8+ thymocytes in Ets-1-deficient mice. J Immunol. 177(2): 905-12.

3. R. Grenningloh, B.Y. Kang, and I.C. Ho. 2005. Ets-1, a functional cofactor of T-bet, is essential for Th1 inflammatory responses. J Exp Med. 201(4): 615-26.

4. S. Eyquem, K. Chemin, M. Fasseu, and J.C. Bories. 2004. The Ets-1 transcription factor is required for complete pre-T cell receptor function and allelic exclusion at the T cell receptor beta locus. Proc Natl Acad Sci U S A. 101(44): 15712-7.

5. S. Eyquem, K. Chemin, M. Fasseu, M. Chopin, F. Sigaux, A. Cumano, and J.C. Bories. 2004. The development of early and mature B cells is impaired in mice deficient for the Ets-1 transcription factor. Eur J Immunol. 34(11): 3187-96.

6. K. Barton, N. Muthusamy, C. Fischer, C.N. Ting, T.L. Walunas, L.L. Lanier, and J.M. Leiden. 1998. The Ets-1 transcription factor is required for the development of natural killer cells in mice. Immunity. 9(4): 555-63.

7. N. Muthusamy, K. Barton, and J.M. Leiden. 1995. Defective activation and survival of T cells lacking the Ets-1 transcription factor. Nature. 377(6550): 639-42.

8. J.E. Levy, O. Jin, Y. Fujiwara, F. Kuo, and N.C. Andrews. 1999. Transferrin receptor is necessary for development of erythrocytes and the nervous system. Nat Genet. 21(4): 396-9.

9. R.J. Jackson, R.W. Engelman, D. Coppola, A.B. Cantor, W. Wharton, and W.J. Pledger. 2003. p21Cip1 nullizygosity increases tumor metastasis in irradiated mice. Cancer Res. 63(12): 3021-5.

10. J. Brugarolas, R.T. Bronson, and T. Jacks. 1998. p21 is a critical CDK2 regulator essential for proliferation control in Rb-deficient cells. J Cell Biol. 141(2): 503-14.

11. J.M. Salvador, M.C. Hollander, A.T. Nguyen, J.B. Kopp, L. Barisoni, J.K. Moore, J.D. Ashwell, and A.J. Fornace, Jr. 2002. Mice lacking the p53-effector gene Gadd45a develop a lupus-like syndrome. Immunity. 16(4): 499-508.

12. C. Deng, P. Zhang, J.W. Harper, S.J. Elledge, and P. Leder. 1995. Mice lacking p21CIP1/WAF1 undergo normal development, but are defective in G1 checkpoint control. Cell. 82(4): 675-84.

13. S.D. Bamforth, J. Braganca, C.R. Farthing, J.E. Schneider, C. Broadbent, A.C. Michell, K. Clarke, S. Neubauer, D. Norris, N.A. Brown, R.H. Anderson, and S. Bhattacharya. 2004. Cited2 controls left-right patterning and heart development through a Nodal-Pitx2c pathway. Nat Genet. 36(11): 1189-96.

14. S.D. Bamforth, J. Braganca, J.J. Eloranta, J.N. Murdoch, F.I. Marques, K.R. Kranc, H. Farza, D.J. Henderson, H.C. Hurst, and S. Bhattacharya. 2001. Cardiac malformations, adrenal agenesis, neural crest defects and exencephaly in mice lacking Cited2, a new Tfap2 co-activator. Nat Genet. 29(4): 469-74.

15. D. Wang, J.M. Pascual, H. Yang, K. Engelstad, X. Mao, J. Cheng, J. Yoo, J.L. Noebels, and D.C. De Vivo. 2006. A mouse model for glut-1 haploinsufficiency. Hum Mol Genet. 15: 1169-1179.

16. J.D. West, J.H. Flockhart, J. Peters, and S.T. Ball. 1990. Death of mouse embryos that lack a functional gene for glucose phosphate isomerase. Genet Res. 56(2-3): 223-36.

17. S. Merkle and W. Pretsch. 1992. A glucosephosphate isomerase (GPI) null mutation in Mus musculus: evidence that anaerobic glycolysis is the predominant energy delivering pathway in early post-implantation embryos. Comp Biochem Physiol B. 101(3): 309-14.

18. B.C. Zingg, W. Pretsch, and H.W. Mohrenweiser. 1995. Molecular analysis of four ENU induced triosephosphate isomerase null mutants in Mus musculus. Mutat Res. 328(2): 163-73.

19. A. Brafman, I. Mett, M. Shafir, H. Gottlieb, G. Damari, S. Gozlan-Kelner, V. Vishnevskia-Dai, R. Skaliter, P. Einat, A. Faerman, E. Feinstein, and T. Shoshani. 2004. Inhibition of oxygen-induced retinopathy in RTP801-deficient mice. Invest Ophthalmol Vis Sci. 45(10): 3796-805.

20. M. Pantaleon, M.B. Harvey, W.S. Pascoe, D.E. James, and P.L. Kaye. 1997. Glucose transporter GLUT3: ontogeny, targeting, and role in the mouse blastocyst. Proc Natl Acad Sci U S A. 94(8): 3795-800.

21. S. Heikkinen, M. Pietila, M. Halmekyto, S. Suppola, E. Pirinen, S.S. Deeb, J. Janne, and M. Laakso. 1999. Hexokinase II-deficient mice. Prenatal death of homozygotes without disturbances in glucose tolerance in heterozygotes. J Biol Chem. 274(32): 22517-23.

22. T.M. Magin, R. Schroder, S. Leitgeb, F. Wanninger, K. Zatloukal, C. Grund, and D.W. Melton. 1998. Lessons from keratin 18 knockout mice: formation of novel keratin filaments, secondary loss of keratin 7 and accumulation of liver-specific keratin 8-positive aggregates. J Cell Biol. 140(6): 1441-51.

23. M. Hesse, T. Franz, Y. Tamai, M.M. Taketo, and T.M. Magin. 2000. Targeted deletion of keratins 18 and 19 leads to trophoblast fragility and early embryonic lethality. Embo J. 19(19): 5060-70.

24. C.T. Culiat, M.L. Klebig, Z. Liu, H. Monroe, B. Stanford, J. Desai, S. Tandan, L. Hughes, M.K. Kerley, D.A. Carpenter, D.K. Johnson, E.M. Rinchik, and Q. Li. 2005. Identification of mutations from phenotype-driven ENU mutagenesis in mouse chromosome 7. Mamm Genome. 16(8): 555-66.

25. S. Merkle, J. Favor, J. Graw, S. Hornhardt, and W. Pretsch. 1992. Hereditary lactate dehydrogenase A-subunit deficiency as cause of early postimplantation death of homozygotes in Mus musculus. Genetics. 131(2): 413-21.

26. J. Chesney, S. Telang, A. Yalcin, A. Clem, N. Wallis, and R. Bucala. 2005. Targeted disruption of inducible 6-phosphofructo-2-kinase results in embryonic lethality. Biochem Biophys Res Commun. 331(1): 139-46.

27. F. Bernassola, M. Federici, M. Corazzari, A. Terrinoni, M.L. Hribal, V. De Laurenzi, M. Ranalli, O. Massa, G. Sesti, W.H. McLean, G. Citro, F. Barbetti, and G. Melino. 2002. Role of transglutaminase 2 in glucose tolerance: knockout mice studies and a putative mutation in a MODY patient. Faseb J. 16(11): 1371-8.

28. P. Carmeliet, V. Ferreira, G. Breier, S. Pollefeyt, L. Kieckens, M. Gertsenstein, M. Fahrig, A. Vandenhoeck, K. Harpal, C. Eberhardt, C. Declercq, J. Pawling, L. Moons, D. Collen, W. Risau, and A. Nagy. 1996. Abnormal blood vessel development and lethality in embryos lacking a single VEGF allele. Nature. 380(6573): 435-9.

29. K. Aase, G. von Euler, X. Li, A. Ponten, P. Thoren, R. Cao, Y. Cao, B. Olofsson, S. Gebre-Medhin, M. Pekny, K. Alitalo, C. Betsholtz, and U. Eriksson. 2001. Vascular endothelial growth factor-B-deficient mice display an atrial conduction defect. Circulation. 104(3): 358-64.

30. D. Bellomo, J.P. Headrick, G.U. Silins, C.A. Paterson, P.S. Thomas, M. Gartside, A. Mould, M.M. Cahill, I.D. Tonks, S.M. Grimmond, S. Townson, C. Wells, M. Little, M.C. Cummings, N.K. Hayward, and G.F. Kay. 2000. Mice lacking the vascular endothelial growth factor-B gene (Vegfb) have smaller hearts, dysfunctional coronary vasculature, and impaired recovery from cardiac ischemia. Circ Res. 86(2): E29-35.
