# Supplementary material for: Maternal diabetes alters transcriptional programs in the developing embryo
Source: BMC Genomics. 2009 Jun 18;10:274. doi: 10.1186/1471-2164-10-274 (PMC2715936; doi:10.1186/1471-2164-10-274)
Supplement: Additional file 4 — References for in vivo function of genes affected in diabetes exposed embryos. The file contains a list of references for the in vivo function of particular genes. [file 1471-2164-10-274-S4.doc]

Additional file 4: **References for in vivo function of genes affected in diabetes exposed embryos.**

| Function | GeneSymbol | Rerefences |
| --- | --- | --- |
|  |  |  |
| embryonic | Abcb7 | 1 |
|  | Adam10 | 2 |
|  | Agtr2 | 3 |
|  | Ap1g1 | 4 |
|  | Aplp2 | 5 |
|  | Bcl11a | 6 |
|  | Creb1 | 7 |
|  | Cxadr | 8, 9 |
|  | Dcx | 10 |
|  | Efnb2 | 11 |
|  | Epha3 | 12 |
|  | Gad1 | 13, 14 |
|  | Grb10 | 15, 16 |
|  | Hif1a | 17, 18 |
|  | Il6st | 19 |
|  | Itgav | 20, 21 |
|  | Kras, | 22, 23 |
|  | Mbtps1 | 24, 25 |
|  | Ndst1 | 26 |
|  | Nedd4 | 27 |
|  | Nsd1 | 28 |
|  | Ogt | 29 |
|  | Pdgfra | 30 |
|  | Ptprs | 31, 32 |
|  | Pxn | 33 |
|  | Scd2 | 34 |
|  | Sema3a | 35, 36 |
|  | Setdb1 | 37 |
|  | Sfrs2 | 38 |
|  | Slc2a1/Glut1ASa | 39, 40 |
|  | Tfrc | 41 |
|  | Tgfbr1 | 42, 43 |
|  | Top2b | 44 |
|  | Twsg1 | 45-48 |
|  | Vcl | 49 |
|  |  |  |
| cardiovascular | Adam10 | 2 |
|  | Agtr2 | 50, 51 |
|  | Cxadr | 8, 9 |
|  | Dysf | 52, 53 |
|  | Epha3 | 12 |
|  | Efnb2 | 11 |
|  | Hif1a | 17 |
|  | Il6st | 19 |
|  | Itgav | 20, 21 |
|  | Pdgfra | 30 |
|  | Pxn | 33 |
|  | Sema3a | 36 |
|  | Sfrs2 | 38 |
|  | Tgfbr1 | 42, 54 |
|  | Vcl | 55 |
|  |  |  |
| neural tube defects | Adam10 | 2 |
|  | Hif1a | 17, 18 |
|  | Pdgfra | 30 |
|  | Tfrc | 41 |
|  | Tgfbr | 42, 54 |
|  | Twsg1 | 45-48 |
|  | Vcl | 49 |
|  |  |  |
| metabolic/growth defect | Ap1g1 | 4 |
|  | Aplp2 | 5 |
|  | Ghr | 56 |
|  | Grb10 | 15, 16 |
|  | Hmga1 | 57 |
|  | Mapk10 | 58 |
|  | Mbtps1 | 24, 25 |
|  | Mtap2 | 59 |
|  | Nedd4 | 27 |
|  | Ptprs | 31, 32 |
|  | Scd2 | 34 |
|  | Tnks2 | 60, 61 |
|  | Top2b | 44 |
|  | Upp1 | 62 |
|  | Zfp385 | 63 |
|  |  |  |
| diabetes | Ghr | 64 |
|  | Hmga1 | 57 |

References:

1. C. Pondarre, B.B. Antiochos, D.R. Campagna, S.L. Clarke, E.L. Greer, K.M. Deck, A. McDonald, A.P. Han, A. Medlock, J.L. Kutok, S.A. Anderson, R.S. Eisenstein, and M.D. Fleming. 2006. The mitochondrial ATP-binding cassette transporter Abcb7 is essential in mice and participates in cytosolic iron-sulfur cluster biogenesis. Hum Mol Genet. 15(6): 953-64.

2. T. Maretzky, K. Reiss, A. Ludwig, J. Buchholz, F. Scholz, E. Proksch, B. de Strooper, D. Hartmann, and P. Saftig. 2005. ADAM10 mediates E-cadherin shedding and regulates epithelial cell-cell adhesion, migration, and beta-catenin translocation. Proc Natl Acad Sci U S A. 102(26): 9182-7.

3. H. Nishimura, E. Yerkes, K. Hohenfellner, Y. Miyazaki, J. Ma, T.E. Hunley, H. Yoshida, T. Ichiki, D. Threadgill, J.A. Phillips, 3rd, B.M. Hogan, A. Fogo, J.W. Brock, 3rd, T. Inagami, and I. Ichikawa. 1999. Role of the angiotensin type 2 receptor gene in congenital anomalies of the kidney and urinary tract, CAKUT, of mice and men. Mol Cell. 3(1): 1-10.

4. D. Zizioli, C. Meyer, G. Guhde, P. Saftig, K. von Figura, and P. Schu. 1999. Early embryonic death of mice deficient in gamma-adaptin. J Biol Chem. 274(9): 5385-90.

5. M. Rassoulzadegan, Y. Yang, and F. Cuzin. 1998. APLP2, a member of the Alzheimer precursor protein family, is required for correct genomic segregation in dividing mouse cells. Embo J. 17(16): 4647-56.

6. P. Liu, J.R. Keller, M. Ortiz, L. Tessarollo, R.A. Rachel, T. Nakamura, N.A. Jenkins, and N.G. Copeland. 2003. Bcl11a is essential for normal lymphoid development. Nat Immunol. 4(6): 525-32.

7. E. Hummler, T.J. Cole, J.A. Blendy, R. Ganss, A. Aguzzi, W. Schmid, F. Beermann, and G. Schutz. 1994. Targeted mutation of the CREB gene: compensation within the CREB/ATF family of transcription factors. Proc Natl Acad Sci U S A. 91(12): 5647-51.

8. A.A. Dorner, F. Wegmann, S. Butz, K. Wolburg-Buchholz, H. Wolburg, A. Mack, I. Nasdala, B. August, J. Westermann, F.G. Rathjen, and D. Vestweber. 2005. Coxsackievirus-adenovirus receptor (CAR) is essential for early embryonic cardiac development. J Cell Sci. 118(Pt 15): 3509-21.

9. D.R. Asher, A.M. Cerny, S.R. Weiler, J.W. Horner, M.L. Keeler, M.A. Neptune, S.N. Jones, R.T. Bronson, R.A. Depinho, and R.W. Finberg. 2005. Coxsackievirus and adenovirus receptor is essential for cardiomyocyte development. Genesis. 42(2): 77-85.

10. J.C. Corbo, T.A. Deuel, J.M. Long, P. LaPorte, E. Tsai, A. Wynshaw-Boris, and C.A. Walsh. 2002. Doublecortin is required in mice for lamination of the hippocampus but not the neocortex. J Neurosci. 22(17): 7548-57.

11. R.H. Adams, G.A. Wilkinson, C. Weiss, F. Diella, N.W. Gale, U. Deutsch, W. Risau, and R. Klein. 1999. Roles of ephrinB ligands and EphB receptors in cardiovascular development: demarcation of arterial/venous domains, vascular morphogenesis, and sprouting angiogenesis. Genes Dev. 13(3): 295-306.

12. A. Vaidya, A. Pniak, G. Lemke, and A. Brown. 2003. EphA3 null mutants do not demonstrate motor axon guidance defects. Mol Cell Biol. 23(22): 8092-8.

13. B.G. Condie, G. Bain, D.I. Gottlieb, and M.R. Capecchi. 1997. Cleft palate in mice with a targeted mutation in the gamma-aminobutyric acid-producing enzyme glutamic acid decarboxylase 67. Proc Natl Acad Sci U S A. 94(21): 11451-5.

14. H. Asada, Y. Kawamura, K. Maruyama, H. Kume, R.G. Ding, N. Kanbara, H. Kuzume, M. Sanbo, T. Yagi, and K. Obata. 1997. Cleft palate and decreased brain gamma-aminobutyric acid in mice lacking the 67-kDa isoform of glutamic acid decarboxylase. Proc Natl Acad Sci U S A. 94(12): 6496-9.

15. K.R. Wick, E.D. Werner, P. Langlais, F.J. Ramos, L.Q. Dong, S.E. Shoelson, and F. Liu. 2003. Grb10 inhibits insulin-stimulated insulin receptor substrate (IRS)-phosphatidylinositol 3-kinase/Akt signaling pathway by disrupting the association of IRS-1/IRS-2 with the insulin receptor. J Biol Chem. 278(10): 8460-7.

16. M. Charalambous, F.M. Smith, W.R. Bennett, T.E. Crew, F. Mackenzie, and A. Ward. 2003. Disruption of the imprinted Grb10 gene leads to disproportionate overgrowth by an Igf2-independent mechanism. Proc Natl Acad Sci U S A. 100(14): 8292-7.

17. L.E. Kotch, N.V. Iyer, E. Laughner, and G.L. Semenza. 1999. Defective vascularization of HIF-1alpha-null embryos is not associated with VEGF deficiency but with mesenchymal cell death. Dev Biol. 209(2): 254-67.

18. V. Compernolle, K. Brusselmans, D. Franco, A. Moorman, M. Dewerchin, D. Collen, and P. Carmeliet. 2003. Cardia bifida, defective heart development and abnormal neural crest migration in embryos lacking hypoxia-inducible factor-1alpha. Cardiovasc Res. 60(3): 569-79.

19. U.A. Betz, W. Bloch, M. van den Broek, K. Yoshida, T. Taga, T. Kishimoto, K. Addicks, K. Rajewsky, and W. Muller. 1998. Postnatally induced inactivation of gp130 in mice results in neurological, cardiac, hematopoietic, immunological, hepatic, and pulmonary defects. J Exp Med. 188(10): 1955-65.

20. W.S. Argraves and C.J. Drake. 2005. Genes critical to vasculogenesis as defined by systematic analysis of vascular defects in knockout mice. Anat Rec A Discov Mol Cell Evol Biol. 286(2): 875-84.

21. B.L. Bader, H. Rayburn, D. Crowley, and R.O. Hynes. 1998. Extensive vasculogenesis, angiogenesis, and organogenesis precede lethality in mice lacking all alpha v integrins. Cell. 95(4): 507-19.

22. L. Johnson, D. Greenbaum, K. Cichowski, K. Mercer, E. Murphy, E. Schmitt, R.T. Bronson, H. Umanoff, W. Edelmann, R. Kucherlapati, and T. Jacks. 1997. K-ras is an essential gene in the mouse with partial functional overlap with N-ras. Genes Dev. 11(19): 2468-81.

23. K. Koera, K. Nakamura, K. Nakao, J. Miyoshi, K. Toyoshima, T. Hatta, H. Otani, A. Aiba, and M. Katsuki. 1997. K-ras is essential for the development of the mouse embryo. Oncogene. 15(10): 1151-9.

24. K.J. Mitchell, K.I. Pinson, O.G. Kelly, J. Brennan, J. Zupicich, P. Scherz, P.A. Leighton, L.V. Goodrich, X. Lu, B.J. Avery, P. Tate, K. Dill, E. Pangilinan, P. Wakenight, M. Tessier-Lavigne, and W.C. Skarnes. 2001. Functional analysis of secreted and transmembrane proteins critical to mouse development. Nat Genet. 28(3): 241-9.

25. J. Yang, J.L. Goldstein, R.E. Hammer, Y.A. Moon, M.S. Brown, and J.D. Horton. 2001. Decreased lipid synthesis in livers of mice with disrupted Site-1 protease gene. Proc Natl Acad Sci U S A. 98(24): 13607-12.

26. K. Grobe, M. Inatani, S.R. Pallerla, J. Castagnola, Y. Yamaguchi, and J.D. Esko. 2005. Cerebral hypoplasia and craniofacial defects in mice lacking heparan sulfate Ndst1 gene function. Development. 132(16): 3777-86.

27. I. Lexicon Genetics, "NIH initiative supporting placement of Lexicon Genetics, Inc. mice into public repositories" MGI Direct Data Submission 2005. 2005. NIH initiative supporting placement of Lexicon Genetics, Inc. mice into public repositories" MGI Direct Data Submission 2005. MGI Direct Data Submission.

28. G.V. Rayasam, O. Wendling, P.O. Angrand, M. Mark, K. Niederreither, L. Song, T. Lerouge, G.L. Hager, P. Chambon, and R. Losson. 2003. NSD1 is essential for early post-implantation development and has a catalytically active SET domain. Embo J. 22(12): 3153-63.

29. R. Shafi, S.P. Iyer, L.G. Ellies, N. O'Donnell, K.W. Marek, D. Chui, G.W. Hart, and J.D. Marth. 2000. The O-GlcNAc transferase gene resides on the X chromosome and is essential for embryonic stem cell viability and mouse ontogeny. Proc Natl Acad Sci U S A. 97(11): 5735-9.

30. M.D. Tallquist and P. Soriano. 2003. Cell autonomous requirement for PDGFRalpha in populations of cranial and cardiac neural crest cells. Development. 130(3): 507-18.

31. M. Elchebly, J. Wagner, T.E. Kennedy, C. Lanctot, E. Michaliszyn, A. Itie, J. Drouin, and M.L. Tremblay. 1999. Neuroendocrine dysplasia in mice lacking protein tyrosine phosphatase sigma. Nat Genet. 21(3): 330-3.

32. M.J. Wallace, J. Batt, C.A. Fladd, J.T. Henderson, W. Skarnes, and D. Rotin. 1999. Neuronal defects and posterior pituitary hypoplasia in mice lacking the receptor tyrosine phosphatase PTPsigma. Nat Genet. 21(3): 334-8.

33. M. Hagel, E.L. George, A. Kim, R. Tamimi, S.L. Opitz, C.E. Turner, A. Imamoto, and S.M. Thomas. 2002. The adaptor protein paxillin is essential for normal development in the mouse and is a critical transducer of fibronectin signaling. Mol Cell Biol. 22(3): 901-15.

34. M. Miyazaki, A. Dobrzyn, P.M. Elias, and J.M. Ntambi. 2005. Stearoyl-CoA desaturase-2 gene expression is required for lipid synthesis during early skin and liver development. Proc Natl Acad Sci U S A. 102(35): 12501-6.

35. S.M. Catalano, E.K. Messersmith, C.S. Goodman, C.J. Shatz, and A. Chedotal. 1998. Many major CNS axon projections develop normally in the absence of semaphorin III. Mol Cell Neurosci. 11(4): 173-82.

36. O. Behar, J.A. Golden, H. Mashimo, F.J. Schoen, and M.C. Fishman. 1996. Semaphorin III is needed for normal patterning and growth of nerves, bones and heart. Nature. 383(6600): 525-8.

37. J.E. Dodge, Y.K. Kang, H. Beppu, H. Lei, and E. Li. 2004. Histone H3-K9 methyltransferase ESET is essential for early development. Mol Cell Biol. 24(6): 2478-86.

38. J.H. Ding, X. Xu, D. Yang, P.H. Chu, N.D. Dalton, Z. Ye, J.M. Yeakley, H. Cheng, R.P. Xiao, J. Ross, J. Chen, and X.D. Fu. 2004. Dilated cardiomyopathy caused by tissue-specific ablation of SC35 in the heart. Embo J. 23(4): 885-96.

39. D. Wang, J.M. Pascual, H. Yang, K. Engelstad, X. Mao, J. Cheng, J. Yoo, J.L. Noebels, and D.C. De Vivo. 2006. A mouse model for glut-1 haploinsufficiency. Hum Mol Genet. 15: 1169-1179.

40. C.W. Heilig, T. Saunders, F.C. Brosius, 3rd, K. Moley, K. Heilig, R. Baggs, L. Guo, and D. Conner. 2003. Glucose transporter-1-deficient mice exhibit impaired development and deformities that are similar to diabetic embryopathy. Proc Natl Acad Sci U S A. 100(26): 15613-8.

41. R.M. Ned, W. Swat, and N.C. Andrews. 2003. Transferrin receptor 1 is differentially required in lymphocyte development. Blood. 102(10): 3711-8.

42. J. Larsson, M.J. Goumans, L.J. Sjostrand, M.A. van Rooijen, D. Ward, P. Leveen, X. Xu, P. ten Dijke, C.L. Mummery, and S. Karlsson. 2001. Abnormal angiogenesis but intact hematopoietic potential in TGF-beta type I receptor-deficient mice. Embo J. 20(7): 1663-73.

43. T. Seki, K.H. Hong, and S.P. Oh. 2006. Nonoverlapping expression patterns of ALK1 and ALK5 reveal distinct roles of each receptor in vascular development. Lab Invest. 86(2): 116-29.

44. X. Yang, W. Li, E.D. Prescott, S.J. Burden, and J.C. Wang. 2000. DNA topoisomerase IIbeta and neural development. Science. 287(5450): 131-4.

45. A. Wills, R.M. Harland, and M.K. Khokha. 2006. Twisted gastrulation is required for forebrain specification and cooperates with Chordin to inhibit BMP signaling during X. tropicalis gastrulation. Dev Biol. 289(1): 166-78.

46. M. Melnick, A. Petryk, G. Abichaker, D. Witcher, A.D. Person, and T. Jaskoll. 2006. Embryonic salivary gland dysmorphogenesis in Twisted gastrulation deficient mice. Arch Oral Biol. 51(5): 433-8.

47. L. Zakin, B. Reversade, H. Kuroda, K.M. Lyons, and E.M. De Robertis. 2005. Sirenomelia in Bmp7 and Tsg compound mutant mice: requirement for Bmp signaling in the development of ventral posterior mesoderm. Development. 132(10): 2489-99.

48. A. Petryk, R.M. Anderson, M.P. Jarcho, I. Leaf, C.S. Carlson, J. Klingensmith, W. Shawlot, and M.B. O'Connor. 2004. The mammalian twisted gastrulation gene functions in foregut and craniofacial development. Dev Biol. 267(2): 374-86.

49. W. Xu, H. Baribault, and E.D. Adamson. 1998. Vinculin knockout results in heart and brain defects during embryonic development. Development. 125(2): 327-37.

50. L. Hein, G.S. Barsh, R.E. Pratt, V.J. Dzau, and B.K. Kobilka. 1995. Behavioural and cardiovascular effects of disrupting the angiotensin II type-2 receptor in mice. Nature. 377(6551): 744-7.

51. T. Inagami and T. Senbonmatsu. 2001. Dual effects of angiotensin II type 2 receptor on cardiovascular hypertrophy. Trends Cardiovasc Med. 11(8): 324-8.

52. K. Wenzel, C. Geier, F. Qadri, N. Hubner, H. Schulz, B. Erdmann, V. Gross, D. Bauer, R. Dechend, R. Dietz, K.J. Osterziel, S. Spuler, and C. Ozcelik. 2007. Dysfunction of dysferlin-deficient hearts. J Mol Med. 85(11): 1203-14.

53. R. Han, D. Bansal, K. Miyake, V.P. Muniz, R.M. Weiss, P.L. McNeil, and K.P. Campbell. 2007. Dysferlin-mediated membrane repair protects the heart from stress-induced left ventricular injury. J Clin Invest. 117(7): 1805-13.

54. S.J. Watkins, L. Jonker, and H.M. Arthur. 2006. A direct interaction between TGFbeta activated kinase 1 and the TGFbeta type II receptor: implications for TGFbeta signalling and cardiac hypertrophy. Cardiovasc Res. 69(2): 432-9.

55. A.E. Zemljic-Harpf, S. Ponrartana, R.T. Avalos, M.C. Jordan, K.P. Roos, N.D. Dalton, V.Q. Phan, E.D. Adamson, and R.S. Ross. 2004. Heterozygous inactivation of the vinculin gene predisposes to stress-induced cardiomyopathy. Am J Pathol. 165(3): 1033-44.

56. F. Lupu, J.D. Terwilliger, K. Lee, G.V. Segre, and A. Efstratiadis. 2001. Roles of growth hormone and insulin-like growth factor 1 in mouse postnatal growth. Dev Biol. 229(1): 141-62.

57. D. Foti, E. Chiefari, M. Fedele, R. Iuliano, L. Brunetti, F. Paonessa, G. Manfioletti, F. Barbetti, A. Brunetti, C.M. Croce, and A. Fusco. 2005. Lack of the architectural factor HMGA1 causes insulin resistance and diabetes in humans and mice. Nat Med. 11(7): 765-73.

58. D.D. Yang, C.Y. Kuan, A.J. Whitmarsh, M. Rincon, T.S. Zheng, R.J. Davis, P. Rakic, and R.A. Flavell. 1997. Absence of excitotoxicity-induced apoptosis in the hippocampus of mice lacking the Jnk3 gene. Nature. 389(6653): 865-70.

59. J. Teng, Y. Takei, A. Harada, T. Nakata, J. Chen, and N. Hirokawa. 2001. Synergistic effects of MAP2 and MAP1B knockout in neuronal migration, dendritic outgrowth, and microtubule organization. J Cell Biol. 155(1): 65-76.

60. S.J. Hsiao, M.F. Poitras, B.D. Cook, Y. Liu, and S. Smith. 2006. Tankyrase 2 poly(ADP-ribose) polymerase domain-deleted mice exhibit growth defects but have normal telomere length and capping. Mol Cell Biol. 26(6): 2044-54.

61. Y.J. Chiang, M.L. Nguyen, S. Gurunathan, P. Kaminker, L. Tessarollo, J. Campisi, and R.J. Hodes. 2006. Generation and characterization of telomere length maintenance in tankyrase 2-deficient mice. Mol Cell Biol. 26(6): 2037-43.

62. D. Cao, J.J. Leffert, J. McCabe, B. Kim, and G. Pizzorno. 2005. Abnormalities in uridine homeostatic regulation and pyrimidine nucleotide metabolism as a consequence of the deletion of the uridine phosphorylase gene. J Biol Chem. 280(22): 21169-75.

63. Y. Kimura, A. Hart, M. Hirashima, C. Wang, D. Holmyard, J. Pittman, X.L. Pang, C.W. Jackson, and A. Bernstein. 2002. Zinc finger protein, Hzf, is required for megakaryocyte development and hemostasis. J Exp Med. 195(7): 941-52.

64. J.E. Rowland, A.M. Lichanska, L.M. Kerr, M. White, E.M. d'Aniello, S.L. Maher, R. Brown, R.D. Teasdale, P.G. Noakes, and M.J. Waters. 2005. In vivo analysis of growth hormone receptor signaling domains and their associated transcripts. Mol Cell Biol. 25(1): 66-77.
