# Supplementary material for: Maternal diabetes alters transcriptional programs in the developing embryo
Source: BMC Genomics. 2009 Jun 18;10:274. doi: 10.1186/1471-2164-10-274 (PMC2715936; doi:10.1186/1471-2164-10-274)
Supplement: Additional file 5 — Primer sequences for quantitative RT-PCR. The file contains a list of RefSeqIDs, position information and sequences for primers to amplify particular genes. The amplification rate for each primer pair is also listed. [file 1471-2164-10-274-S5.doc]

Additional file 5: **Primer sequences for quantitative RT-PCR**

| Gene | RefSeq ID | Forward Primer | | Reverse primer | | Amplification |
| --- | --- | --- | --- | --- | --- | --- |
| symbol |  | starting position | Sequence | starting position | Sequence | rate |
| Adam10 | NM_007399 | 1670 | 5'-AAGCAGTGCAGTCCGAGTCAA-3' | 1812 | 5'-GGATCAGATGCTGGGCAAAG-3' | 1.9 |
| Api5 | NM_007466 | 1460 | 5'-CAAGTTCAGGTTCACCACCTAAGA-3' | 1578 | 5'-CCTCTCATAATTAAAGTTGCTCAGATTG-3' | 1.9 |
| Atrx | NM_009530 | 7278 | 5'-CCCAAGTCCAAGCACTAGCATT-3' | 7421 | 5'-GGAGCCGTCTATTCATAAGTATTCG-3' | 2.0 |
| Baz1b | NM_011714 | 3518 | 5'-GTTTGAGGCCCGGGTCAT-3' | 3632 | 5'-CTGCTTAGGCGCCATAAATCC-3' | 1.9 |
| Cited4 | NM_019563 | 988 | 5'-AGCCTTCGGATTCCACGTT-3' | 1097 | 5'-GTTAGTAGGCTGCTCGAGAAGGA-3' | 1.7 |
| Creb1 | NM_009952 | 661 | 5'-CAAACTAGCAGTGGGCAGTACATT-3' | 741 | 5'-CTGTACCCCATCCGTACCATTG-3' | 2.0 |
| Cxadr | NM_001025192 | 798 | 5'-GAAGTACGAGAAGGAAGTTCATCATGA-3' | 911 | 5'-ATGGATCCCAGGGAGGAATG-3' | 1.7 |
| Dcx | NM_010025 | 1224 | 5'-CAGCAGTCAGCTCTCAACACCTAA-3' | 1356 | 5'-GGAATCGCCAAGTGAATCAGA-3' | 1.8 |
| Efnb2 | NM_010111 | 666 | 5'-AGATGCAAGTTCTGCTGGATCAG-3' | 799 | 5'-TGCCATCGGTGCTAGAACCT-3' | 2.0 |
| Hif1a | NM_010431 | 1299 | 5'-CAGTACAGGATGCTTGCCAAAA-3' | 1423 | 5'-ATACCACTTACAACATAATTCACACACACA-3' | 1.8 |
| Hmga1 | NM_016660 | 120 | 5'-GCTGGTCGGGAGTCAGAAAG-3' | 210 | 5'-GGCGGCGCCCTTATTC-3' | 1.9 |
| Ilst6 | NM_010560 | 2201 | 5'-GTTCCTGATCCTTCCAAGAGTCAT-3' | 2320 | 5'-CACAACGCTTACATCAGTGAAATTG-3' | 2.0 |
| Lin28 | NM_145833 | 2233 | 5'-CTTTGAGATCGCCACAAACCTA-3' | 2332 | 5'-ACAAGAAACCAAGATCAGACACACA-3' | 1.6 |
| Mt2 | NM_008632 | 135 | 5'-GCAAATGTACTTCCTGCAAGAAAA-3' | 229 | 5'-GTCGGAAGCCTCTTTGCAGAT-3' | 1.9 |
| Mtap2 | NM_008630 | 5314 | 5'-GACTTAAGCCATGTGACATCCAAAT-3' | 5416 | 5'-CCTTGAAATCCAGTTTTACACTCTCA-3' | 2.0 |
| Ogt | NM_139144 | 2632 | 5'-ATTAGCAATGGACTGGCGACTAC-3' | 2728 | 5'-CATACTGGGAACGGGTGGTT-3' | 2.0 |
| Pdgfra1 | NM_011058 | 2361 | 5'-GTCCCCATGCTTGAAAGGAA-3' | 2490 | 5'-CATCGTCCGAAAGGAGGTTTT-3' | 1.9 |
| Pdh18 | NM_130448 | 2965 | 5'-GGAAACAAATATTCCAGGAGCTATAGA-3' | 3079 | 5'-GCCCCAAATCATAATCGCTATC-3' | 1.8 |
| Phip | XM_001004343 | 3137 | 5'-GACCGGTGGATCATTTACCAT-3' | 3251 | 5'-GCGGTCACCTATATTCCATCG-3' | 2.0 |
| Pole4 | NM_025882 | 205 | 5'-CGGGACAGGAAGCCATCTT-3' | 282 | 5'-AGCAGTAGGCATCTTTTGCGATA-3' | 2.0 |
| Rod1 | NM_144904 | 483 | 5'-CAGCCTGTTTATATCCAGTATTCCAA-3' | 613 | 5'-GCTCCAGGAAGGGACAAGTTT-3' | 1.6 |
| Sema3a | NM_009152 | 1736 | 5'-AACTATCAGTGGGTGCCTTACCAA-3' | 1844 | 5'-CATCATCAGGAAGGTCCTTTGTG-3' | 2.0 |
| Slc2a1 | NM_011400 | 1441 | 5'-GGGCATGTGCTTCCAGTATGT-3' | 1512 | 5'-ACGAGGAGCACCGTGAAGAT-3' | 2.0 |
| Tfrc | NM_011638 | 1319 | 5'-CCAGTGTGGGAACAGGTCTTC-3' | 1464 | 5'-CAACCACTCAGTGGCACCAA-3' | 1.9 |
| Tgfbr1 | NM_011577 | 1199 | 5'-CGCGCTGACATCTATGCAAT-3' | 1306 | 5'-AGGTACAAGATCATAATAAGGCAACTGA-3' | 2.0 |
| Twsg2 | NM_023053 | 577 | 5'-GCACCACCAAAACGTGTCTGT-3' | 690 | 5'-CACTGGTGGATGGACATGCA-3' | 2.0 |
| Vcl | NM_009502 | 2992 | 5'-TGCCAAGCAGTGCACAGATAA-3' | 3115 | 5'-GGTCCGGCCCAGCATAGT-3' | 1.9 |
